# ‘Small’ Technology, Big Power: Micropore Engineering for High‐Performance Flow Battery Membranes

**DOI:** 10.1002/smll.202513508

**Published:** 2026-02-18

**Authors:** Chunhua Wei, Wenbin Fan, Yue Luo, Nannan Jia, Chuzhang Hong, Jieyu Yan, Xinhua Liu, Rui Tan

**Affiliations:** ^1^ School of Resources Environment and Materials Guangxi University Nanning China; ^2^ Department of Chemical Engineering Swansea University Swansea UK; ^3^ School of Transportation Science and Engineering Beihang University Beijing China

**Keywords:** ion transport mechanism, membrane optimisation, microporous and functional membranes, redox flow batteries, selectivity and conductivity

## Abstract

Achieving carbon neutrality demands large‐scale deployment of renewable energy, which in turn requires efficient, durable, and low‐cost electrochemical energy storage systems. Redox flow batteries (RFBs) have emerged as a leading technology for grid‐scale storage owing to their decoupled power and energy, long cycle life, and intrinsic safety. At the heart of RFB performance lies the membrane, which governs ion transport, selectivity, stability, and overall system cost. Optimizing membrane properties is therefore central to advancing RFB technology. This Review examines recent progress in flow battery membranes, emphasizing their working mechanisms, performance criteria, and key challenges. We discuss the structural characteristics, ion transport behavior, and modification strategies of diverse membrane types, including ion‐exchange membranes, non‐ion‐exchange membranes, porous membranes, and emerging functional materials such as covalent organic frameworks, metal–organic frameworks, and polymers of intrinsic microporosity. Particular attention is given to strategies that enhance selectivity and ionic conductivity through synergistic effects, such as size exclusion, Donnan exclusion, and dielectric regulation. Finally, we outline future directions for membrane design, including multi‐mechanism coupling, sub‐nanometer pore engineering, defect modulation, and composite functionalization, providing a framework for developing high‐performance, low‐cost, and long‐life membranes for next‐generation flow batteries.

## Graphical abstract

This review systematically analyzes recent advances in redox flow batteries (RFBs) membrane separators, focusing on the shift from traditional ion‐exchange membranes to emerging functional porous membranes: Covalent Organic Frameworks (COFs), Metal‐Organic Frameworks (MOFs), and Polymers of Intrinsic Microporosity (PIMs). It discusses ion transport, selectivity, challenges, and modification strategies, and proposes forward‐looking design principles for next‐generation high‐performance, low‐cost, durable membranes.

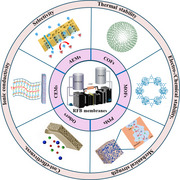

## 1 Overview

### 1.1 Electrochemical Energy Storage

The pursuit of carbon neutrality, coupled with rising energy demand and fossil fuel‐related environmental stress, is accelerating the transition toward renewable energy technologies such as solar and wind power [1, 2, 3, 4, 5, 6]. Over the past decade, renewable energy has expanded rapidly, securing an increasingly prominent role in the global energy landscape. According to the International Energy Agency (IEA), renewables accounted for approximately 30% of global electricity generation in 2022, with solar photovoltaics and wind power contributing more than 80% of this total. Looking ahead, renewable energy is poised to become a dominant pillar of the world's energy supply [7]. However, the inherent intermittency and variability of renewable energy sources [8], together with fluctuations in grid demand, result in spatiotemporal mismatches that limit their direct and efficient utilization. Large‐scale energy storage technologies with high energy density, long cycle life, and scalability are therefore essential. By storing and releasing electricity in a controlled and timely manner, such systems provide vital flexibility for the large‐scale integration of renewables and underpin the low‐carbon transformation of the global energy system [9].

Energy storage technologies can be categorized into five types based on their energy conversion mechanisms: *Mechanical Energy Storage (MES)* achieves storage through the mutual conversion between electrical energy and mechanical potential energy/kinetic energy, represented by pumped storage hydro (PSH), compressed air energy storage (CAES), and flywheel energy storage (FES) [10], which are suitable for large‐scale peak shaving and short‐term response scenarios [11]; *Electromagnetic Energy Storage (EMES)* utilizes the characteristics of electromagnetic fields, featuring extremely fast response speed, with supercapacitors (SCs) and superconducting magnetic energy storage (SMES) being applicable for high‐power output and power grid stability control [12, 13]; *Thermal Energy Storage (TES)* stores energy in the form of sensible heat, latent heat, or chemical heat, and is widely used in solar thermal power generation, building heating, and waste heat recovery [14]; *Chemical Energy Storage* (based on chemical bonds) realizes energy storage through fuel synthesis and conversion, with hydrogen energy and synthetic fuels being suitable for long‐cycle cross‐industry energy coupling [15]; as one of the most widely used categories, *Electrochemical Energy Storage (EES)* stores chemical energy relying on electrochemical reactions between electrodes and electrolytes. Its core technologies include lithium‐ion batteries (LIBs) [16, 17, 18], redox flow batteries (RFBs) [19], sodium‐based batteries (SBBs) [20], and lead‐acid batteries (LABs) [21], etc. Among them, LIBs dominate the fields of new energy vehicles and distributed energy storage due to their advantages of high energy density and high charge–discharge efficiency, while RFBs have become a core choice for long‐duration large‐scale energy storage by virtue of their characteristics such as decoupled capacity and power, and long cycle life. Various technologies complement each other in different application scenarios, fully covering diversified energy storage needs.

Among the various energy storage technologies currently available, batteries demonstrate significant technological competitiveness due to their high energy conversion efficiency, rapid response capabilities, flexible deployment, and modular scalability. Based on different technical pathways and material systems, battery systems are applicable across an extremely wide range of scenarios. For large‐scale energy storage, they can be adapted to grid‐level applications, with output power ranging from 1 kW to 10 MW [22, 23]. Although all types of batteries share common advantages, they each feature distinctive technical architectures, operating mechanisms, and performance metrics while facing different sets of challenges. Among these, flow batteries stand out in the field of large‐scale energy storage due to their unique electrochemical energy storage mechanism, long‐duration storage potential, and system scalability, emerging as a key technological direction that overcomes the limitations of traditional batteries.

RFBs have emerged as a preferred technology for large‐scale long‐duration energy storage, thanks to their core advantages of excellent safety performance, long cycle life, and flexible adjustability of power and capacity [24, 25, 26]. As one of the core components of RFBs, the performance of the membrane can determine the coulombic efficiency (CE), energy efficiency (EE), and operational stability of the battery, exerting a crucial impact on the improvement of the battery's comprehensive performance and cost control. Currently, attention to membrane design is continuously rising, but it is important to note that the cross‐membrane permeation of redox‐active species is prone to causing battery capacity fading and performance degradation [27]. Therefore, the development of high‐performance membranes has become a core challenge and key research focus for breaking through technical bottlenecks in the relevant research field.

### 1.2 Membrane Separators in RFBs

**FIGURE 1:**
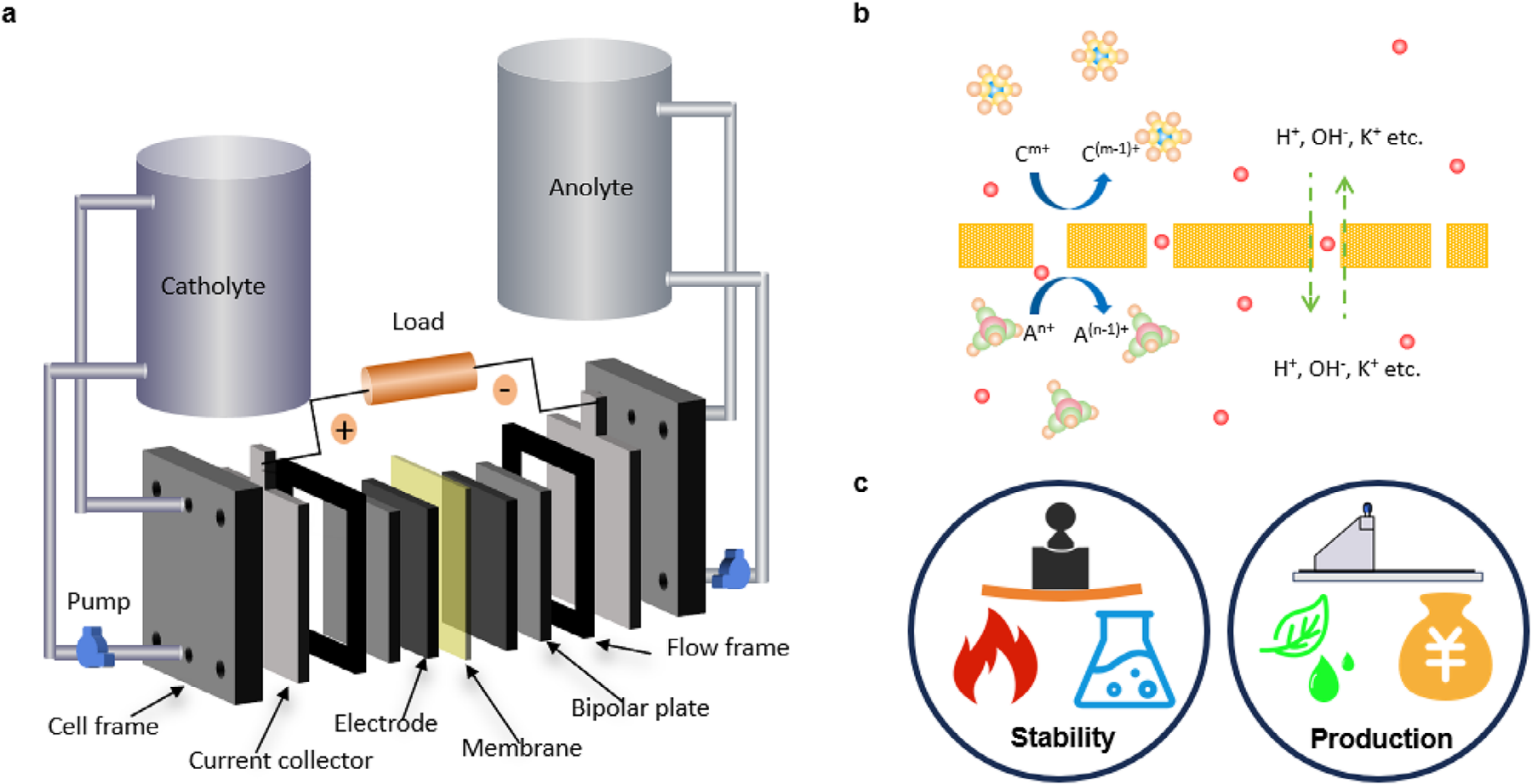
(a) Schematic diagram of a typical RFB. (b) Diagram of the function of an ion‐conductive and molecular‐selective membrane. Anolyte: A^(n‐1)+/n+^ and Catholyte: C^(m‐1)+/m+^. (c) Requirements for RFB membranes, including mechanical strength, thermal stability, chemical stability, ease of manufacturing, low cost, and environmental friendliness.

RFBs systems are composed of core components including positive and negative electrolyte tanks, current collectors, electrodes, and ion‐conducting membranes (Figure 1a). As a key component, the membrane must fulfill dual functions: it needs to effectively block the cross‐compartment permeation of redox‐active species while ensuring the selective high‐flux transport of target ions (Figure 1b), and this characteristic directly determines battery performance [28, 29]. Ideal membrane materials are required to meet multiple stringent criteria: a balance between high ionic conductivity and selectivity, excellent stability (mechanical stability, chemical stability, thermal stability), scalable processability, environmental friendliness, and controllable cost (Figure 1c). Although perfluorinated Nafion membranes have become the mainstream commercial products due to their comprehensive performance, their high cost and limited sieving capability for redox‐active species such as vanadium ions still restrict the commercialization process and cost optimization potential of RFBs systems [30, 31].

In recent years, with the growing attention to the cost‐effectiveness of RFBs, the development of membranes to replace expensive perfluorinated materials has become a research hotspot. Significant efforts have been devoted to non‐perfluorinated cation exchange membranes (CEMs), anion exchange membranes (AEMs), non‐ionic membranes, and porous polymer membranes. However, it remains challenging to develop membrane materials that fully meet the stringent requirements of RFBs. Many candidate membranes suffer from issues such as limited ion selectivity, high area resistance, and insufficient mechanical strength or chemical stability [28]. To address these problems, researchers have explored various modification technologies: for example, incorporating specific functional groups (e.g., quaternary ammonium groups/sulfonic acid groups) into polymers through molecular design and functionalization to enhance the conductivity of specific electrolytes [32, 33]; utilizing composite materials such as inorganic fillers (e.g., TiO_2_, SiO_2_, or graphene) to improve stability and reduce ion permeability [34, 35], and polymer interpenetrating networks (PINs) (e.g., polyether ether ketone (PEEK) and polysulfone (PSf) [36] to balance mechanical strength and ionic conduction; adopting porous membranes combined with size sieving technology, realizing physical blocking of macromolecular redox‐active species through micropore regulation (e.g., composites with polymers of intrinsic microporosity (PIMs), covalent organic frameworks (COFs), and metal–organic frameworks (MOFs)) while allowing the passage of small ions. Through technological iteration, researchers have progressively broken through the bottlenecks of traditional membrane materials via systematic microstructural engineering (such as microphase separation, microporosity construction, and dual‐channel design) and chemical modification (such as functional group modulation, crosslinking, and branching) [37], propelling the development of RFB membranes toward lower cost, higher selectivity, and longer service life.

### 1.3 Inspiration from LIB Separator Design: Engineering Smaller Pores

As is known, practical separators for LIBs must meet a series of stringent performance requirements, including chemical and electrochemical stability, good wettability, appropriate thickness, high mechanical strength, high porosity with pore sizes compatible with carrier ion dimensions, and excellent thermal stability [38, 39, 40]. Early LIB separators were typically fabricated from materials such as polyethylene (PE) or polypropylene (PP) via microporous formation technology, resulting in a porous structure with pore sizes ranging from approximately 0.2 to 1 µm [41, 42]. These micropores can prevent the contact between active material particles on both sides of the electrodes while allowing the free migration of small Li^+^, thereby forming ion transport pathways. However, as battery technology advances toward higher energy density and enhanced safety, traditional microporous separators struggle to meet the comprehensive performance demands of next‐generation batteries [43]. To address this challenge, researchers have successfully refined the pore size of separators to the sub‐nanometer scale (<1 nm) by introducing nanopore technology, ceramic coatings, and functional crosslinkers [44]. This refined pore engineering “from micrometers to nanometers” not only significantly improves the cycle life and rate capability of batteries but also provides a feasible technical pathway for achieving efficient size exclusion in thin‐film separators.

Similarly, beyond the fundamental requirements shared with LIB separators, such as appropriate thickness, mechanical robustness, wettability, and thermal stability, membranes for RFBs must additionally enable both rapid and selective ion transport. Achieving this balance is essential for ensuring high energy efficiency while preventing the crossover of redox‐active species, thereby maintaining long cycle life. Introducing controlled microporosity within RFB membranes offers a promising pathway to meet these dual demands. This review highlights emerging membrane design strategies centered on microporous materials with sub‐nanometer pore structures, tailored to the specific requirements of flow battery systems. By summarizing and analyzing recent advances, we elucidate the intrinsic structure–property–function relationships that drive membrane performance, offering design principles for next‐generation membranes in high‐performance electrochemical energy storage technologies.

## 2 RFBs and Developed Membrane Separators

### 2.1 System Concepts and Working Principles

Based on the different electrolyte solutions used, flow batteries can be classified into two main types: aqueous flow batteries and non‐aqueous flow batteries. Aqueous flow batteries use aqueous solutions as the electrolyte solvent, which has high safety and low cost, but is limited by the electrolysis of water, resulting in a low voltage window. By contrast, non‐aqueous flow batteries rely on organic solvents such as acetonitrile or carbonate esters, or even ionic liquids. These media extend the voltage range and raise energy density, though the flammability of organics inevitably introduces safety concerns. According to their structure, flow batteries can be divided into conventional flow batteries, solid–liquid hybrid flow batteries, gas–liquid hybrid flow batteries, and innovative solar flow batteries that combine solar cells and flow batteries [27].

In the conventional structured RFBs, the active materials of the positive and negative electrodes are stored independently in external tanks in the form of liquid electrolytes, and then circulated to the battery stack via a pumping system for electrochemical reactions. This design allows the energy capacity (dependent on the volume and concentration of the electrolyte) and power (dependent on the size of the battery stack) to be designed and scaled independently, thus achieving a high degree of flexibility and scalability. The VRFB is one of the most mature and highly commercialized representatives among them [45].

Hybrid structures are multiphase systems composed of redox‐active liquid electrolytes combined with redox‐active solid metals or gaseous substances. Such systems not only retain the core characteristic of decoupling of power and capacity of traditional RFBs but also integrate the prominent advantage of high energy density of metal/gaseous systems [46]. Zinc is an ideal choice due to its negative standard potential, fast reaction kinetics, low‐cost, high‐energy density, and abundant reserves. Since the advent of the first zinc/bromine semi‐solid RFB [47], zinc‐based chemistry has attracted considerable attention in the field of hybrid systems by virtue of its aforementioned advantages. Circulating redox‐active liquid substances or supplementing fresh electrolytes can effectively solve the problem of byproduct deposition on electrodes/metal surfaces in traditional static metal–air devices, thereby reducing the loss of active area and the reduction in reaction rate. However, limited by factors such as high catalyst cost, uncontrollable metal dendrite growth, insufficient stability of redox‐active substances, and poor performance of membrane materials, the comprehensive performance and operational safety of hybrid batteries still need further optimization [48].

**FIGURE 2:**
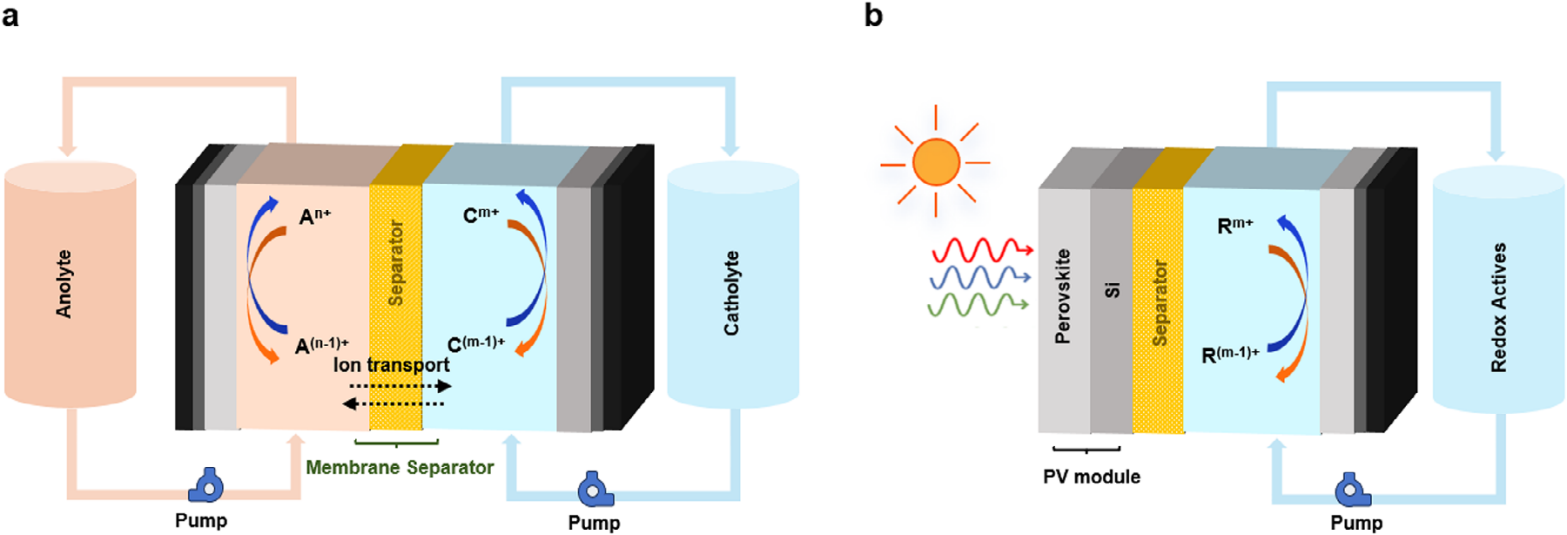
Schematics showing the basic configuration and working principles of RFBs. (a) a typical RFB. (b) a combined PV‐RFB system.

Innovative structures refer to the integration of advantageous features from different energy storage technologies. A promising example is the solar flow battery (SFBs) [49] (Figure 2b). By leveraging photoelectrochemical coupling, it directly converts light energy into chemical energy stored in the liquid electrolyte through photoelectrochemical reactions, achieving direct storage from light to chemical energy. Since the energy carrier is a liquid, storage duration can be easily extended by increasing the volume of liquid tanks, offering high scalability. However, its light‐to‐electricity conversion efficiency remains relatively low. The development of solar flow batteries is still at the proof‐of‐concept stage and faces numerous technological challenges.

### 2.2 Redox Chemistries for RFBs

Redox‐active chemicals are the fundamental basis for achieving the mutual conversion between electrical energy and chemical energy in RFBs, governing key performance metrics such as the charge/discharge processes, energy density, and cycling stability. The performance of redox‐active species is determined by four core parameters [50]: Redox Potential—The potential difference between the positive and negative electrode active materials directly determines the output voltage of the battery; a larger potential difference theoretically leads to higher energy density. Solubility—The solubility of active materials in the electrolyte determines the energy storage capacity of the electrolyte; higher solubility enables more energy to be stored per unit volume. Stability—This includes chemical stability (to avoid self‐decomposition, cross‐linking, or contamination) and electrochemical stability (to ensure reversible valence changes during charge/discharge cycles). Molecular Size—This affects the cross‐membrane migration and permeation of active materials; larger molecules can effectively prevent crossover contamination. According to the type of materials, redox chemistry can be classified into three categories: metal ion and halide ion chemistry, metal ligand complexes chemistry, and non‐metallic organic redox chemistry.

#### 2.2.1 Metal‐Ion and Halogen‐Ion Redox Chemistry

This category represents the earliest and most mature branch of RFB technology, with transition metal ions (e.g., V, Fe, Cr, and Zn) or halogen ions (e.g., Br, I) serving as the core redox‐active centers [26, 51, 52]. Its fundamental redox mechanism involves the reversible valence change of metal ions or the redox conversion of halogen ions. The reaction process is straightforward and controllable, without requiring complex molecular structural reconstruction. Key advantages include stable redox potentials, rapid electron transfer kinetics, and strong chemical stability, making it the mainstream choice for commercially deployed RFBs. The most representative system, the all‐VRFBs, is the most mature commercial RFB technology. Since the positive and negative electrolytes share the same active element, it avoids the capacity imbalance caused by electrolyte crossover. However, vanadium electrolytes are costly [53], and the toxicity of vanadium species has raised concerns [54], as electrolyte leakage could lead to environmental pollution. Furthermore, the crossover of small‐sized ions such as V^2+^/V^3+^ and VO_2_^+^/VO_2_^+^ through the membrane poses challenges to the cycling performance and service life of VRFBs [55]. To address these limitations, researchers have been actively developing alternative RFB systems.

Zinc‐based RFBs are regarded as highly promising energy storage solutions due to their inherent advantages, such as high energy density, high open‐circuit voltage, and low cost. The high redox potential of zinc electrodes, coupled with the abundant availability and environmental friendliness of zinc resources, makes zinc a preferred anode active material for many hybrid RFB systems [56]. However, their application remains limited by issues such as zinc dendrite growth, side reactions, and ion crossover, which can lead to short circuits, capacity degradation, and safety concerns [57]. Current research primarily focuses on developing dendrite‐resistant membranes, optimizing electrolytes, and modifying electrodes to improve the cycle stability and safety of the batteries.

Polysulfide‐based flow batteries are a class of energy storage technology that relies on the reversible redox reactions between sulfur (typically in the form of polysulfide ions S_x_^2−^) and a paired substance (such as bromine/bromide ions, iron‐based materials, etc.). Their core advantages lie in the low cost and abundant resources of the active materials, as well as their high theoretical energy density. Although the traditional polysulfide/bromine system offers certain benefits, it faces challenges such as the strong corrosiveness and volatility of bromine, along with severe crossover contamination. Replacing bromine with iron‐based materials as the positive electrode active material effectively mitigates these drawbacks. The ferrocyanide/ferricyanide redox couple ([Fe(CN)_6_]^4−^/[Fe(CN)_6_]^3−^) has emerged as a highly promising positive electrode active material in this system due to its high stability, low toxicity, and cost‐effectiveness [58]. While the sulfur‐iron system addresses the key issues associated with bromine, common challenges of polysulfide flow batteries remain, including crossover contamination between polysulfide ions and the positive electrode active material, related side reactions, and sluggish reaction kinetics of polysulfide electrodes [59]. Therefore, current research focuses on developing highly selective membranes, optimizing electrolytes, and improving electrode interfaces to overcome these bottlenecks.

#### 2.2.2 Metal–Ligand Complex and Organic Redox Materials

Compared to traditional metal‐ion systems, the core advantage of redox‐active organic materials (ROMs) lies in the flexible designability of their molecular structures, enabling the tunability of redox potential, solubility, and stability. For example, the N‐alkyl‐carboxylate‐functionalized anthraquinone derivative DAEAQ exhibits a solubility of 0.99 m in alkaline electrolyte, and the electron‐donating nature of its N‐alkyl groups lowers the redox potential to −0.61 V versus SHE, which is beneficial for increasing the full‐cell voltage [60]. Similarly, through asymmetric modification of tetrathiafulvalene (TTF) by introducing polyethylene glycol (PEG) chains and a perfluorophenyl (PerF) group, the second redox potential can be raised to 0.65 V versus Ag/Ag^+^ while improving solubility, thereby significantly enhancing the voltage of non‐aqueous batteries [61]. Such molecular engineering strategies endow ROMs with the potential for achieving high energy density and long cycle life. For instance, the zwitterionic naphthalene diimide (CBu)_2_NDI maintains 100% capacity retention after 5070 cycles at a concentration of 0.1 m in a neutral aqueous system [62]. ROMs also feature wide raw material sources, low cost, and environmental friendliness. The total electrolyte cost for a full battery combining (CBu)_2_NDI synthesized via the atmospheric pressure method with K_4_Fe(CN)_6_ can be as low as 6.58$/Ah. Furthermore, designing large‐sized molecules or utilizing intermolecular interactions can effectively suppress active species crossover through the membrane. The molecular dimension of (CBu)_2_NDI (3.28 nm) exceeds the channel width of Nafion 212 membrane (2.4 nm). Relying on steric hindrance and electrostatic repulsion, its permeability remains below 2.6 × 10^−14 ^cm^2 ^s^−1^, providing an example for constructing highly stable “crossover‐free” systems [62]. Nevertheless, ROMs still face critical challenges in practical applications, including chemical degradation, viscosity increase at high concentrations, long‐term cycling stability, and scalable synthesis.

#### 2.2.3 Selection of Redox Species

The chemical stability and molecular size of redox species are key factors influencing the cycle stability and operational lifespan of batteries. Chemical stability directly determines whether the species can maintain structural integrity and reaction reversibility during long‐term charge–discharge cycles, thereby avoiding capacity decay caused by decomposition, side reactions, or agglomeration. Meanwhile, the molecular size is closely related to the migration behavior of the species through the ion‐exchange membrane. An appropriate size can effectively suppress crossover contamination, mitigate membrane aging and capacity loss, thus ensuring efficient and durable operation of the battery. The use of environmentally friendly redox‐active electrolytes can further mitigate the environmental footprint of membrane production. Therefore, for specific battery systems, the rational design and screening of redox species with both high stability and suitable molecular dimensions are crucial pathways to achieving long cycle life and high cost‐effectiveness in flow batteries.

### 2.3 Membranes for RFBs

#### 2.3.1 Key Parameters of RFBs

Ionic conductivity, ionic selectivity, structural stability, scalability, and cost‐effectiveness together constitute the core performance evaluation system for RFB membranes. These key indicators fundamentally constrain the comprehensive operational efficiency of RFBs. For RFB membranes, their capacity to conduct charge carriers such as H^+^, Li^+^, Na^+^, K^+^, and OH^−^ must be optimized to ensure the full exertion of the battery's overall performance. As the core parameter for measuring this conduction capacity, ionic conductivity directly determines the overall internal resistance of the flow battery, thereby exerting a crucial impact on voltage efficiency (VE), energy efficiency (EE), and output power density [63]. Meanwhile, high selectivity of the membrane material toward redox‐active species is also indispensable — this property can significantly suppress the transmembrane permeation of active species, providing robust support for the long‐term cycling stability of RFBs.

The stability of membrane materials is another key factor ensuring the long‐term reliable operation of RFBs. In practical applications, RFBs are often exposed to harsh operating conditions such as strong acidic/alkaline electrolytes, highly oxidizing metal ions, internal pressure fluctuations, and metal dendrite penetration, all of which may cause structural degradation and performance attenuation of membrane materials. Therefore, RFB membranes targeting industrial applications need to meet multiple performance requirements: they must not only possess sufficient mechanical strength to withstand pressure shocks during system operation but also maintain chemical structural stability in electrolyte systems with a wide pH range, while exhibiting excellent electrochemical resistance to strong oxidizing substances.

##### 2.3.1.1 Ion Conductivity

**FIGURE 3:**
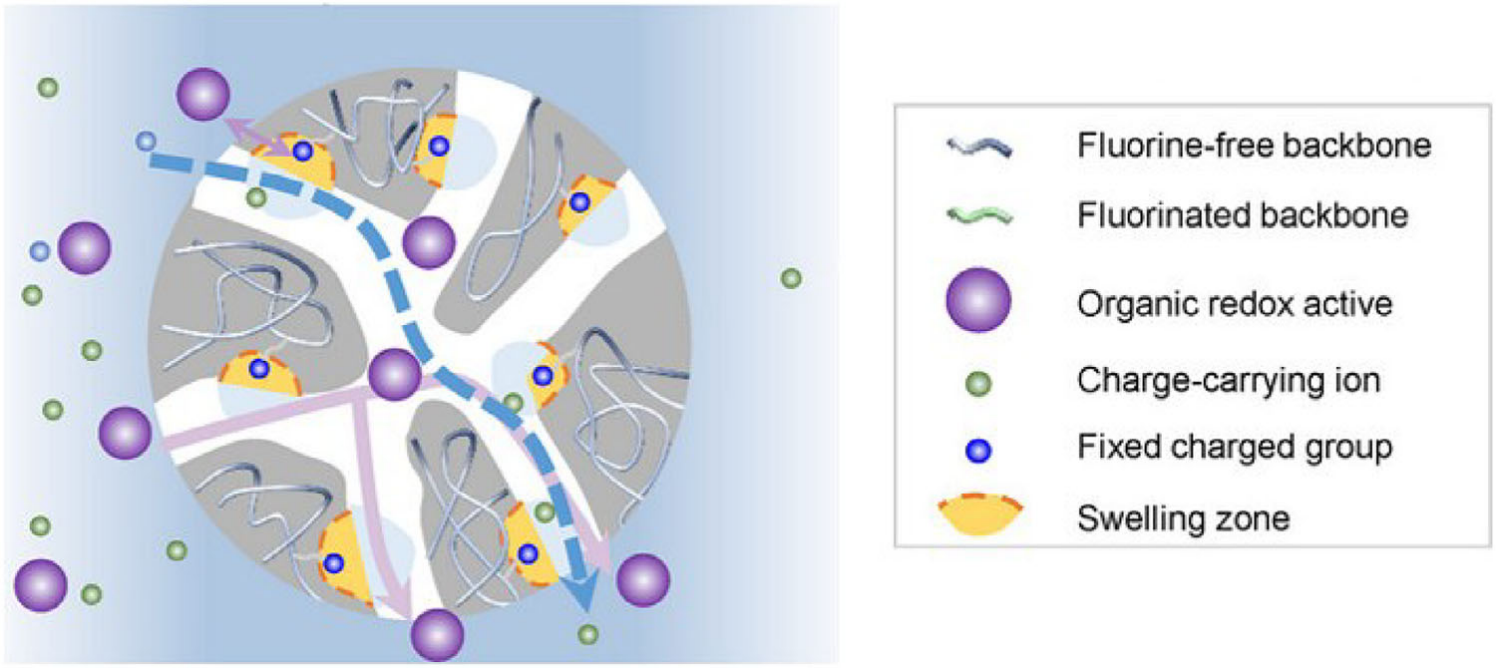
Schematic illustrations showing the existing IEMs for RFBs [65]. Reproduced with permission [65]. Copyright 2025, Wiley‐VCH GmbH.

The ion transport mechanisms in ion‐exchange membranes (IEMs) primarily encompass the ion‐exchange mechanism, the vehicle mechanism, and the Grotthuss mechanism. The ion‐exchange mechanism relies on reversible interactions between fixed ionic functional groups within the membrane and counterions, facilitating ion‐hopping migration between adjacent sites. The vehicle mechanism involves the overall diffusion of ions in their hydrated forms within the solvent‐filled channels. The Grotthuss mechanism describes proton diffusion through hydrogen‐bond networks in water clusters, which mainly consists of two coordinated steps: proton hopping and water molecule reorientation, enabling proton transfer from a donor site to an adjacent acceptor. These microscopic transport mechanisms collectively determine the macroscopic ionic conductivity of the membrane. As an intrinsic property of membrane materials, ionic conductivity directly regulates the transport process and dynamic behavior of ions within the membrane. Its driving force stems from the coupled effects of concentration gradient diffusion, electric field migration, and fluid convection. For aqueous electrolyte systems, constructing dedicated ion channels inside the membrane is an effective strategy to enhance ionic conductivity; this approach can significantly accelerate the migration of charge carriers, thereby strengthening the overall conductive performance of the membrane. For instance, introducing hydrophilic ionic groups into membrane materials can substantially improve ion transport efficiency. These groups can be divided into two categories based on their charge properties: negatively charged groups include ─SO_3_^−^, ─PO_3_^2−^, ─PO_3_H^−^, ─COO^−^, etc.; positively charged groups consist of ─NH_3_^+^, ─NR_2_H^+^, ─NR_3_^+^, etc. However, it should be noted that while traditional ion‐exchange membranes can significantly boost ionic conductivity, they often induce excessive membrane swelling and exacerbate the permeation of active species (Figure 3). Therefore, in the process of optimizing ionic conductivity, it is imperative to simultaneously consider the permeation of redox species within the membrane and achieve the balanced regulation of both properties.

Controllable microstructures are equally crucial for enhancing ionic conductivity while suppressing the permeability of redox‐active substances. PSFA membranes consist of hydrophilic sulfonic acid groups through narrow water channels with a diameter of about 1 nanometer. During the membrane preparation process, the assembly of hydrophilic side chains and hydrophobic backbones induces microphase separation, forming hydrophilic regions with water cluster structures upon hydration [64]. These spontaneously formed hydrophilic regions in PSFA membranes not only provide more nanoscale space for ion transport but also selectively transmit ions or redox species through interconnected bottleneck structures. These narrow ion channels can prevent the permeation of certain large‐sized redox‐active species. However, Nafion, as the most mature and highly commercialized representative among PSFA membranes [29], remains troubled by electrolyte crossover issues and fails to meet current demands, thus requiring further improvement.

Over the past few years, researchers have conducted extensive studies on the synthesis of hydrocarbon‐based ion exchange membranes, with the core focus on the controllable regulation of phase‐separated microstructures and the optimal design of pore structures. Among these approaches, the partial functionalization of hydrophilic groups or side chains in materials is a common method to induce phase separation, as exemplified by sulfonated polyphenylene [66], sulfonated polyetheretherketone (S‐PEEK) [67], etc. However, the introduction of a single hydrophilic functional group makes it difficult to precisely regulate the hydrophilicity of the membrane. Therefore, researchers have adopted a modification strategy of combining hydrophobic and hydrophilic groups (e.g., simultaneous introduction of sulfonic acid groups and alkyl hydrophobic side chains onto polybenzimidazole (PBI) [68]), which can control membrane swelling and achieve high ionic conductivity with limited water uptake. In addition, the pore structure and its size distribution also significantly affect ion transport performance. The construction of pores is mainly realized through methods such as phase inversion, template agent introduction, or utilization of non‐solvent polymers [69, 70]. Nevertheless, these methods tend to form large‐sized pores, thereby exacerbating the problem of transmembrane permeation of redox species.

##### 2.3.1.2 Selectivity

**FIGURE 4:**
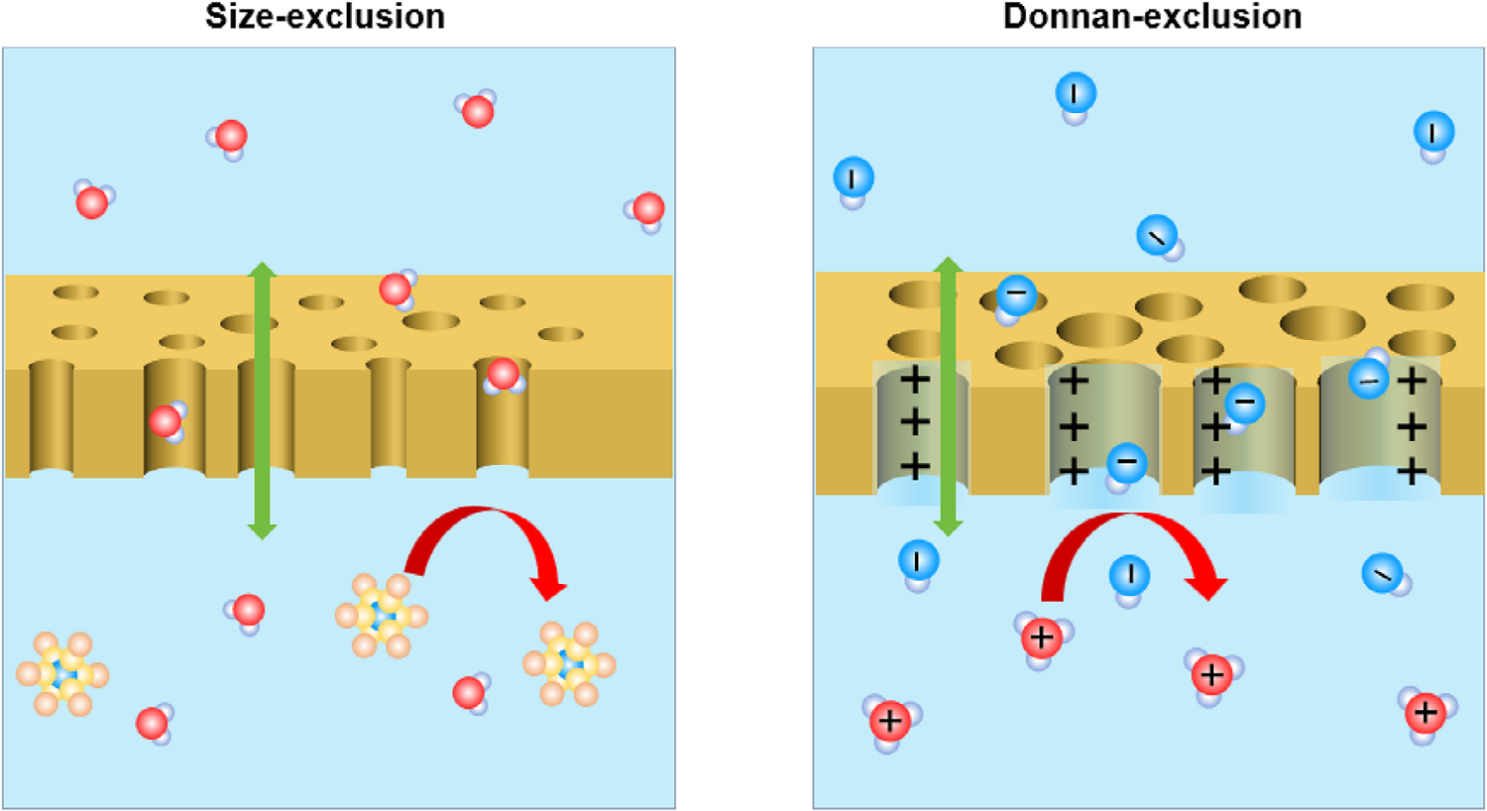
Schematics showing the working principles of size‐exclusion and Donnan‐exclusion.

Selectivity is a core attribute that determines the current efficiency and service life of RFBs. As mentioned previously, the process of optimizing ionic conductivity often leads to a simultaneous increase in the permeability of redox species. Therefore, the core requirement for high‐performance membranes is to enhance ionic conductivity while blocking the crossover of redox‐active species and ensuring the selective transport of charge carriers. To achieve this performance balance, it is necessary to implement precise regulation of the membrane’ s microstructure (e.g., constructing nanoscale ion channels) and charge characteristics (e.g., introducing functional groups with repulsive effects) (Figure 4). This ultimately extends the cycle life of the battery and maintains the stability of battery efficiency.

Size‐exclusion refers to the membrane's selective restriction of larger ions based on physical pore size, thereby achieving the selective transport of small ions such as protons. This function can be realized by constructing precisely sized pores or channels through regulating material structure and preparation processes (e.g., controlling polymer phase separation, utilizing porous materials, introducing crosslinking networks, and leveraging acid‐base pair interactions). For example, By regulating the topological structures of supramolecular side chains (linear, branched, cyclic), the pseudo‐nanophase separation structures within the membrane are precisely controlled. The interconnected hydrophilic channels (∼12 nm) formed by cyclic side chains effectively block vanadium ions while enhancing proton conductivity [71]. The two‐dimensional MFI‐type zeolite membrane, relying on its regular pores of approximately 0.55 nm, achieved efficient proton conduction and effective blocking of vanadium ions, significantly improving the ion selectivity of flow batteries [72]. In situ hypercrosslinking of quaternized polyphenylene oxide (QPPO) membranes can construct an HC‐QPPO network structure with sub‐nanometer micropores (<1 nm), which allows efficient transport of small ions such as Na^+^ and Cl^−^ while strongly rejecting large‐sized redox‐active molecules like BTMAP‐Vi, thereby significantly enhancing ion selectivity and battery cycling stability [73]. Additionally, by adjusting the ratio of quaternized polyphenylene oxide to SPEEK, the interaction of “acid‐base pairs” can compress the water channel size, forming ion channels with controllable size and high selectivity [74].

Donnan‐exclusion is another mechanism for membrane selectivity. Its principle lies in the charged membrane electrostatically repelling species of the same charge while facilitating the transport of counterions (Figure 4). For instance, the fluorinated poly(aryl piperidinium) anion exchange membrane (PFDP) constructed by incorporating quaternary ammonium groups forms positively charged channels within the membrane, effectively inhibiting the transmembrane migration of positively charged vanadium ions [75]. Similarly, the SPEEK membrane with a high degree of sulfonation utilizes sulfonic acid groups (negatively charged) within the membrane to effectively block the transmembrane migration of iodide ions via the Donnan exclusion effect, thereby enhancing cycling stability [76]. Ion conductivity and selectivity are of crucial importance, as mentioned above. Additionally, stability, mechanical strength, cost‐effectiveness, and sustainability are also very important for the development of practical membranes.

#### 2.3.2 Fabrication Methods for RFB Membranes

The microstructure of a membrane (e.g., pore size, porosity, pore connectivity, phase‐separation morphology) fundamentally determines its ion conductivity, selectivity, and mechanical stability. The formation of these structures is highly dependent on the fabrication process. A systematic understanding of membrane fabrication methods is crucial for material design, performance optimization, and eventual large‐scale industrialization. Currently, the fabrication methods for flow battery membranes can be primarily categorized as follows:

##### 2.3.2.1 Phase Inversion Method

Phase inversion is the most common method for forming porous structures by controlling the transition of a polymer solution from a liquid to a solid state, with non‐solvent‐induced phase separation (NIPS) being the most prevalent technique [77]. This method involves casting a polymer solution into a film and then immersing it in a non‐solvent bath (typically water or alcohol). The exchange between the solvent and non‐solvent causes thermodynamic phase separation of the polymer, solidifying it into a membrane. By precisely controlling parameters such as polymer concentration, solvent/non‐solvent type, coagulation bath composition, and temperature, it is possible to fabricate membranes with either symmetric sponge‐like structures or asymmetric finger‐like/pore skin‐support layer structures [78, 79, 80, 81]. For instance, employing a delayed phase separation strategy can promote the formation of a dense, ultra‐thin selective skin layer and a highly porous sublayer, thereby achieving both high selectivity and low area‐specific resistance simultaneously. The NIPS process is relatively simple and easily scalable, making it a mainstream technique for preparing porous polymer membranes.

##### 2.3.2.2 Solution Casting Method

For traditional dense ion exchange membranes (e.g., Nafion, SPEEK), the solution casting method is commonly used. This method involves dissolving the polymer in a suitable solvent to form a homogeneous solution, which is then cast onto a smooth substrate using a doctor blade to form a wet film. Subsequently, a precisely controlled drying process removes the solvent, ultimately forming a dense, non‐porous membrane. The challenge of this method lies in controlling the solvent evaporation rate to avoid pinholes and defects and to obtain a uniform membrane structure.

##### 2.3.2.3 Preparation of Mixed Matrix Membranes

Mixed matrix membranes (MMMs) combine the high processability of a polymer matrix with the unique properties of functional fillers (e.g., MOFs, COFs, inorganic nanoparticles) [82, 83, 84]. Their preparation typically involves first uniformly dispersing the fillers in a polymer solution, followed by membrane formation using the aforementioned phase inversion or solution casting methods. The key challenge is addressing the interfacial compatibility between the fillers and the polymer matrix to prevent the formation of non‐selective defects at the interface. Surface modification of the fillers or the use of in situ growth techniques is often required to achieve strong bonding and uniform distribution of the fillers within the matrix.

##### 2.3.2.4 Interfacial Polymerization and Thin‐Film Composite Membrane Fabrication

The design concept of thin‐film composite (TFC) membranes has been introduced into the flow battery field to minimize mass transfer resistance while ensuring high selectivity [85]. A TFC membrane typically consists of a porous support layer and an ultra‐thin yet dense selective separation layer [86]. Interfacial polymerization (IP) is a classic technique for constructing this selective layer. It involves a polymerization reaction between monomers at the interface of two immiscible solvents, instantaneously forming an extremely thin polyamide or another polymer layer [87]. This method can create separation layers with sub‐nanometer sieve channels, significantly enhancing membrane selectivity, while maintaining proton conductivity due to its ultra‐thin nature.

#### 2.3.3 Types of RFB Membranes

**FIGURE 5:**
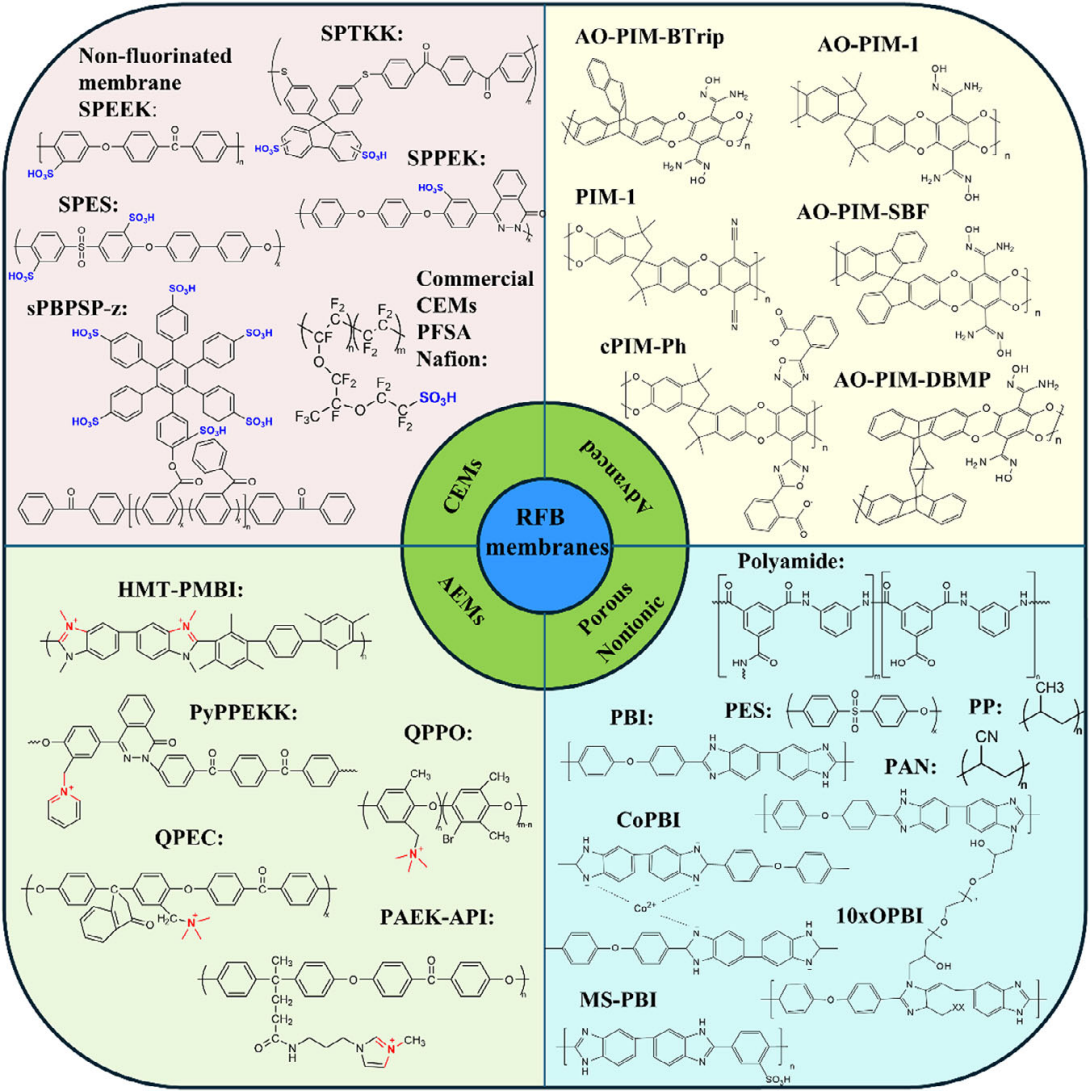
Summary of recent progress on RFB membranes, including CEMs, AEMs, Nonionic membranes, porous membranes, and next‐generation membranes. sPBPSP‐z [66], SPTKK [88], SPPEK [89], sPEEK‐Trip [90], SPPO [91], HMT‐PMBI [92], PyPPEKK [93], PAEK‐API [94], QPPO [91], QPEC [95], AO‐PIM‐1, AO‐PIM‐SBF, AO‐PIM‐DBMP, AO‐PIM‐BTrip [96], cPIM‐Ph [97], PIM‐1 [98], Polyamide [99], PBI [100], S‐OPBI [101], CoPBI [102], 10xOPBI [103], MS‐PBI [100].

The membranes of flow batteries can be classified into four types: IEMs, non‐ion‐exchange membranes, porous membranes, and advanced functional membranes (which have both pore structure and ion functional group properties). Currently, the commercialized flow battery stacks mainly use IEMs, including CEMs, AEMs, amphoteric ion exchange membranes (AIEMs), and embedded ion exchange membranes (MIEMs) (Figure 5).

##### 2.3.3.1 Cation Exchange Membranes

**FIGURE 6:**
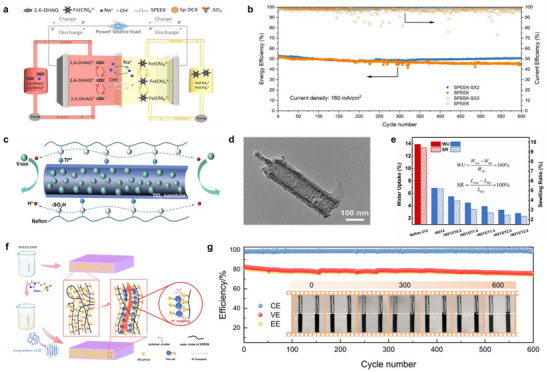
(a) Diagram of an alkaline 2,6‐DHAQ|K_4_Fe(CN)_6_ ORFB. (b) The cell performance of SPEEK and SPEEK‐SX2 at a current density of 160 mA cm^−2^ [104]. (c) TiO_2_ nanotubes embedded in the Nafion matrix. (d) Transmission electron microscope (TEM) image of TiO_2_ nanotubes. (e) The water uptake (WU) and swelling ratio (SR) of different membranes [106]. (f) Schematic Representation of the Preparation Procedure of the SPEEK and Him‐pS Composite Membranes. (g) At a current density of 250 mA cm^−2^, the battery performance of the VRFB assembled using the SPEEK/Him‐pS‐3 composite membrane [111]. (a,b) Reproduced with permission [104]). Copyright 2025, Wiley‐VCH GmbH. (c–e) Reproduced with permission [106]. Copyright 2020, Wiley‐VCH. (f,g) Reproduced with permission [111]. Copyright 2021, American Chemical Society.

CEMs achieve directional transport of cations (e.g., H^+^, K^+^, Na^+^, and Li^+^) and attract counterions via cation exchange functional groups (such as ─SO_3_^−^), but their broad application is constrained by the high cost of commercial membranes like Nafion. To reduce costs, an effective strategy is the incorporate inexpensive organic or inorganic fillers into CEMs, which improves economic viability while preserving performance. For example, adding sulfonated polydichlorooxyethylene (Sp‐DCX) as an organic filler into a sulfonated poly(ether ketone) (SPEEK) matrix improves membrane stability [104], enabling stable operation at a high current density of 160 mA cm^−2^ for 600 cycles with a capacity decay as low as 0.00935% per cycle (Figure 6a,b). In addition to organic fillers, inorganic fillers such as ZrO_2_, TiO_2_, SiO_2_, GO, zeolite, and ceramics are widely used, significantly enhancing mechanical strength, suppressing swelling, and improving ion selectivity [27, 105]. For instance, a hybrid membrane fabricated by incorporating superhydrophilic TiO_2_ nanotubes into a Nafion matrix exhibits low water uptake, low swelling ratio, and good chemical stability (Figure 6c–e). When applied in all‐VRFBs, it demonstrates an energy efficiency of approximately 84.4% and maintains nearly unchanged performance over more than 1400 charge–discharge cycles [106]. Furthermore, organic fillers such as polyvinyl alcohol, polypyrrole, polyaniline, and zwitterionic Nafion‐*g*‐PSBMA have also been used to modify Nafion membranes [107, 108, 109, 110]. Although fillers offer cost advantages, their chemical stability in strong acid or alkali environments remains inadequate, making it difficult to meet the stringent requirements for long‐term operation of membrane materials in flow batteries.

Furthermore, researchers are developing new cationic exchange polymers through post‐sulfonation or copolymerization methods. However, due to the difficulty in precisely controlling the grafting sites and the sulfonation degree of the sulfonic acid groups, the preparation process is highly dependent on precise process regulation. At the same time, these sulfonated polymers have limited chemical stability under extreme conditions such as strong acids/alkalis, making them difficult to operate stably for a long time. Polymer blending technology provides a simpler and more reliable approach for the development of high‐performance and high‐stability membrane materials. By means of blending different polymers, the intrinsic advantages of each component are fully integrated, thereby achieving a synergistic balance between the mechanical properties of the matrix material and the chemical stability of the functional components. For example, blending ether‐free sulfonated polytriphenyl (SPTP) with PBI constructs an internal network through acid‐base interactions between them. The resulting SPTP/PBI blend membrane exhibits excellent chemical stability in strongly acidic and oxidizing environments, and can operate stably for more than 1500 cycles in VRFBs, demonstrating the great potential of the polymer blending strategy in improving membrane durability under extreme electrochemical environments [112]. The blending strategy can also optimize the ion transport path by regulating the nanoscale structure within the membrane. For instance, a study introduced additives containing sulfonic acid and imidazole groups into SPEEK, utilizing the “acid‐base pair” effect formed by them, to promote the directional arrangement of SPEEK molecular chains [111], thereby constructing high‐conductivity nanochannels throughout the membrane (Figure 6f). This structure significantly improves proton conductivity while also enhancing selectivity for vanadium ions, achieving a synergistic improvement in conductivity and selectivity. After 600 cycles, no significant degradation was observed in the composite membrane, and no notable imbalance between the positive and negative electrolytes occurred, demonstrating good long‐term cycling stability in VRFB applications (Figure 6g).

##### 2.3.3.2 Anion Exchange Membranes

**FIGURE 7:**
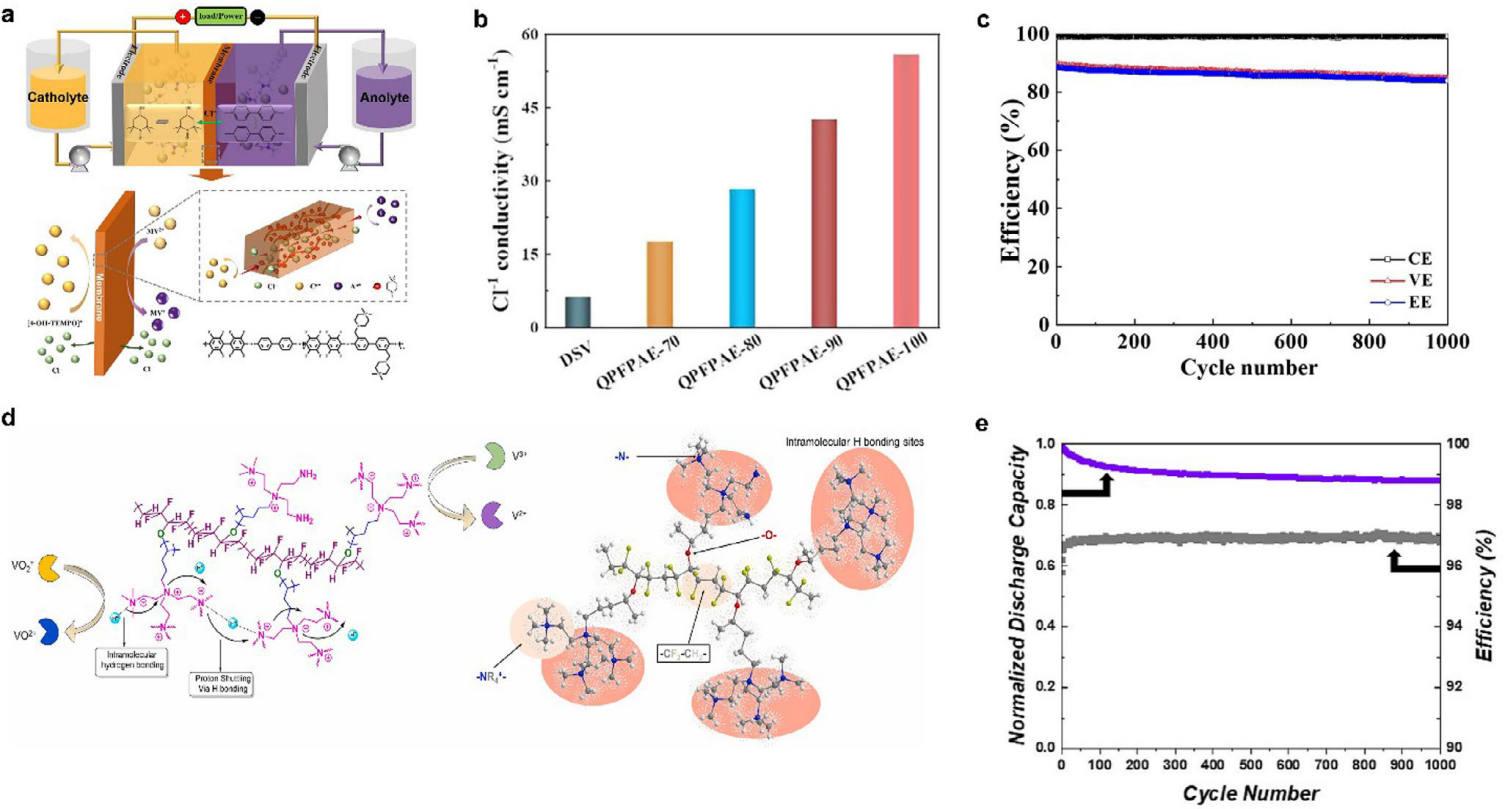
(a) Schematic illustration of the MV^2+^/4‐HO‐TEMPO NAORFB and Cl^−^ conduction mechanism of the QPFPAE AEM. (b) Cl^−^ conductivity of DSV and QPFPAE‐x membranes. (c) Cycling performance at 60 mA cm^−2^ of the QPFPAE‐100 membrane [113]. (d) Schematic Diagram of Miktoarm Anion Exchange Architecture [114]. (e) The capacity (purple) and efficiency (gray) versus cycle for 1000 cycles for TMA‐PE‐6 [115]. (a–c) Reproduced with permission [113]. Copyright 2022, Elsevier B.V. (d) Reproduced with permission [114]. Copyright 2025, Elsevier Ltd. (e) Reproduced with permission [115]. Copyright 2022, American Chemical Society.

AEMs contain anion exchange groups such as quaternary ammonium (─NR_3_^+^), imidazolium, benzimidazolium, and pyridinium. These positively charged groups not only provide transport sites for charge carriers (e.g., OH^−^, Cl^−^, and SO_4_^2−^) but also block the permeation of positively charged redox‐active species (e.g., vanadium ions) via the Donnan exclusion effect. The introduction of anion exchange groups has emerged as a new strategy to suppress vanadium ion crossover in VRFBs. ─NR_3_ is the primary functional group in AEMs. For example, ozone pretreatment followed by grafting 4‐bromo‐1‐butene onto the PVDF backbone leverages the robust C–F backbone for mechanical stability [114], while the quaternary ammonium groups impart desirable electrochemical properties (Figure 7d). A hydrophobic fluorinated poly(arylene ether) backbone coupled with hydrophilic piperazinium groups yields a membrane material with high Cl^−^ conductivity [113], demonstrating exceptional stability and top‐tier performance metrics over 1000 charge–discharge cycles in neutral aqueous organic flow batteries (Figure 7a–c). Additionally, trimethylammonium‐functionalized polyethylene membranes in non‐aqueous RFBs achieved an average capacity retention of 99.99% per cycle, with an overall capacity retention of 88% over 1000 charge/discharge cycles (Figure 7e), exhibiting low crossover and solving the issue of membrane swelling in organic solvents while maintaining high counterion (PF_6_^−^) conductivity.

##### 2.3.3.3 Other IEMs

Functional amphoteric IEMs have attracted increasing attention from researchers due to their combined advantageous characteristics of CEMs and AEMs. Amphoteric IEMs integrate cation and anion exchange groups at the molecular level, which can synergistically improve their ionic conductivity and selectivity. For example, a zwitterionic membrane TA‐SPBP was prepared by introducing sulfonic acid groups and tertiary amino groups onto an ether‐free polybiphenyl backbone through superacid‐catalyzed polycondensation and side chain grafting [116]. The sulfonic acid groups provide proton transport channels to ensure high proton conductivity; the protonated tertiary amino groups effectively block vanadium ion migration through the Donnan exclusion effect. The acid‐base interaction between the two also enhances the dimensional stability and chemical stability of the membrane, thereby simultaneously achieving high ionic selectivity and long cycle life. Additionally, introducing amphoteric groups (e.g., sulfobetaine methacrylate) into the Nafion matrix can synergistically improve its ionic conductivity and selectivity [107].

##### 2.3.3.4 Nonionic Membranes and Porous Separators

**FIGURE 8:**
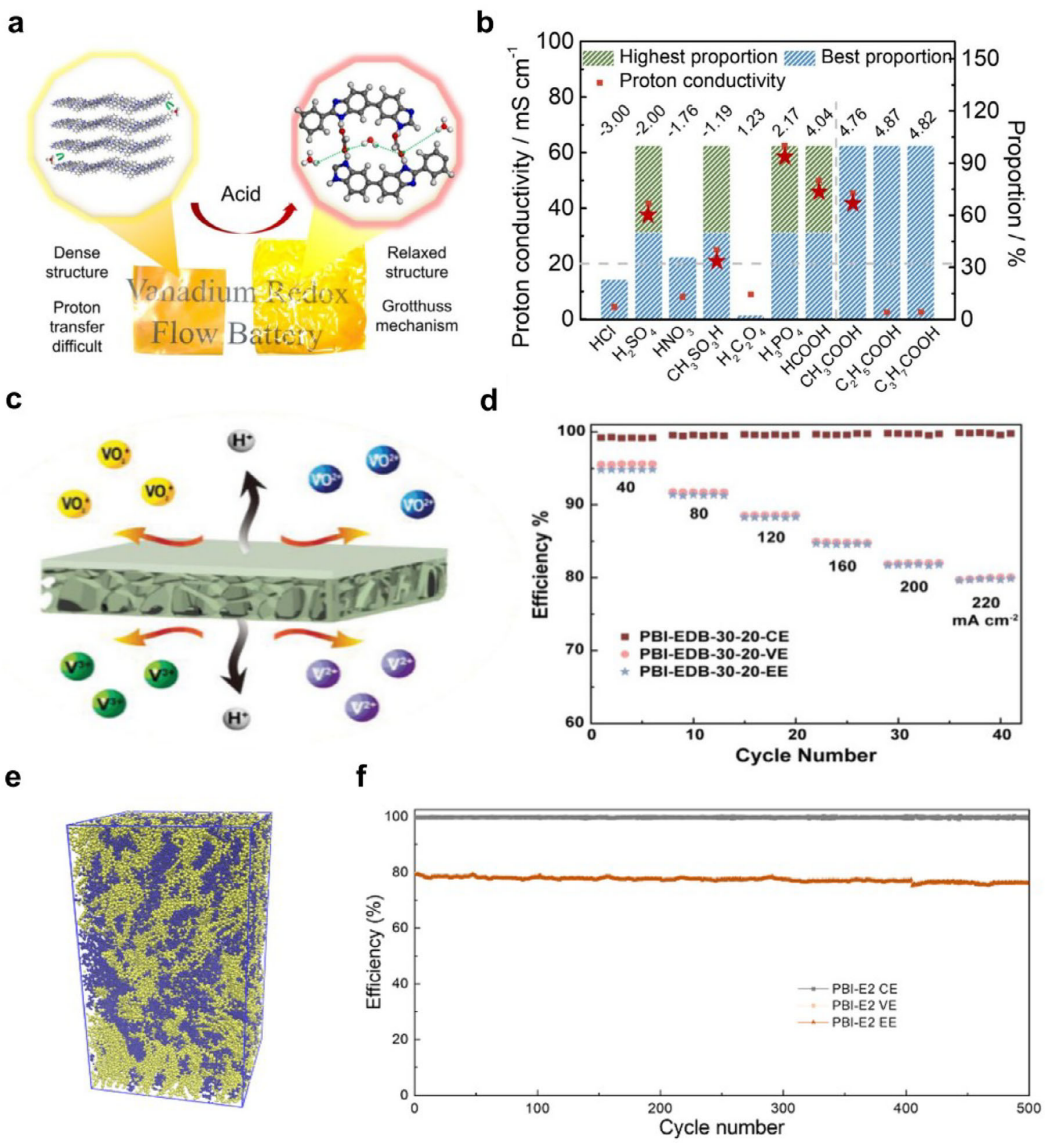
(a) Schematic diagram of the acid swelling strategy for PBI membranes. (b) The highest concentration of different acids, the best proton conductivity of derived PBI membranes, and the corresponding concentration of different acids [118]. (c) The membrane prepared by the two‐step NIPS method. (d) VFB performance of PBI‐EDB‐30‐20 membranes at different current densities (40–220 mA cm^−2^) at ambient temperature [119]. (e) Equilibrium snapshot of the PBI/TEA system from MD simulations. (f) Efficiencies of PBI‐E2‐based vanadium RFB over cycling at 240 mA cm^−2^ [120]. (a,b) Reproduced with permission [118]. Copyright 2025, Elsevier B.V. (c,d) Reproduced with permission [119]. Copyright 2020, Wiley‐VCH GmbH. (e,f) Reproduced with permission [120]. Copyright 2024, Elsevier B.V.

Apart from the charged groups, the functionalized non‐ionic groups provide another approach for developing conductive and highly selective membranes. Such groups include amino (─NH_2_), amine, benzimidazole, polyether, and hydroxyl (─OH). In an acidic environment, the amino groups are easily protonated and form hydrogen bond networks, enhancing the Grotthuss mechanism. Taking the acid expansion PBI strategy as an example, the performance of HCOOH‐PBI is significantly improved compared to ordinary PBI. Its proton conductivity increases from 1.93 to 50 mS cm^−1^ (Figure 8a,b).

Introducing pore structures into non‐ionic membranes is an effective method to enhance the absorption rate of electrolytes and the ionic conductivity. For instance, by using the two‐step non‐solvent‐induced phase separation (NIPS) method to regulate the diffusion rates inside and outside, a polybenzimidazole (PBI) porous membrane with a thin selective layer and a highly porous supporting layer structure was prepared (Figure 8c), achieving simultaneous improvement in ionic selectivity and proton conductivity. At a current density of 220 mA cm^−2^ in a vanadium flow battery, the energy efficiency reached 80% (Figure 8d). The “pseudo‐nanoparticle separation” structure effectively blocks the penetration of vanadium ions while also enhancing the proton conductivity. For example, triethanolamine (TEA) was non‐covalently grafted onto the PBI polymer. The continuous hydrophobic phase formed by the densely packed PBI chains serves as the primary barrier against vanadium ion penetration, with additional electrostatic repulsion provided by the Donnan effect from protonated benzimidazole motifs within the matrix. At the same time, the presence of large‐sized hydrophilic domains and the hydrogen‐bond network extending through the hydrophobic matrix significantly enhances the proton conductivity (Figure 8e). The vanadium flow battery based on this membrane still maintains an energy efficiency of over 80% at a high current density of 240 mA cm^−2^ and exhibits excellent cycle stability with a capacity decay rate of less than 0.04% per cycle after 500 cycles (Figure 8f). Moreover, various porous membranes (such as commercial Celgard, Daramic membranes, as well as PP, PAN, PVDF, PES porous membranes, and COF [117] membranes) have also been explored for use in RFBs.

#### 2.3.4 Next‐Generation of Ion‐Selective Membranes

In recent years, with the continuous development of functional microporous materials, new membrane systems based on functional microporous materials have become a research hotspot due to their unique structural designability. Through precise pore size control and functional modification, such materials have shown great potential in battery membranes. The core preparation substrates of intrinsic microporous membranes mainly cover three categories: MOFs, COFs, and PIMs. Due to their inherent brittleness, MOFs usually need to be combined with flexible polymer supports or binders to form membranes [121]. For example, by combining MOF materials with polymers such as PVDF‐HFP, self‐supporting membranes can be formed, which are applied to battery separators to improve ion transport and battery performance [122]. For COFs, integrating ionic functional groups such as spiroborate bonds [82, 83] or quaternized amino groups [123] into their framework structures is expected to make them efficient ion transport media. For instance, connecting quaternary ammonium cations to the framework main body through flexible ether bond alkyl side chains enables a large number of ordered and uniformly distributed cationic groups in the COF channels, significantly improving anion conduction efficiency [124]. However, the types of functional ionic monomers for COFs are still scarce, and the development of new ionic monomers will be a key direction to expand the application boundaries of COF materials.

In contrast to MOFs and COFs, PIMs are composed of macromolecules with rigid and structurally stable backbones, possessing excellent solution processability and enabling large‐scale membrane fabrication. Due to the high rigidity and twisted structure of their backbones, these polymers exhibit loose molecular chain packing, allowing the formation of sub‐nanoscale micropores with sizes ranging from 0.2 to 0.8 nm. Thus, they can theoretically achieve efficient ion sieving—permitting the passage of small‐sized ions while blocking larger redox‐active species. The combination of the Donnan exclusion effect and the size sieving effect has become an important research direction for designing highly selective flow battery membranes. For instance, sulfonated polyoxanthene membranes with intrinsic microporosity and ionic conductivity demonstrate high ionic conductivity and excellent selectivity for redox‐active anions [125]. These functionalized designs further expand the application potential of PIMs in the field of electrochemical energy storage.

### 2.4 Limitations and Evaluation of Membranes in RFBs

In the research and development field of membranes for RFBs, remarkable achievements have been made in relevant research efforts. Although diversified material systems have opened up multiple feasible pathways for the innovative upgrading of membranes, it still remains a formidable challenge to develop membrane products that can fully meet the requirements of all performance metrics. Regulating multiple properties of membranes—such as thickness, ionic conductivity, pore structure, and polymer chemical properties—is a highly complex systematic issue. There are intricate intrinsic correlations among the various performance characteristics of membranes; optimizing and improving one performance indicator may lead to the decline of other related efficiency indicators. For example, although the introduction of charged groups can enhance ionic conductivity, it may impair the mechanical properties of functionalized membranes, and the interaction between charged groups and redox species can induce membrane fouling. While crosslinking treatment can strengthen mechanical strength and inhibit active species diffusion, reduced electrolyte uptake will concurrently decrease ionic conductivity. Constructing mesoporous/macroporous structures and thinning the membrane layer can effectively lower the surface resistance of RFBs and improve output power, yet it exacerbates the crossover of active species, resulting in reduced capacity retention and shortened cycle life. The core research challenge at the current stage lies in how to achieve precise matching between membranes and the electrochemical environment of RFB systems through the synergistic regulation of their various properties.

## 3 Functional Porous Membranes for Electrochemical Applications

Porous materials play a crucial functional role in electrochemical devices, particularly in the field of functional electrolyte separators, which has now become a research hotspot with significant progress. Some typical porous materials are constructed from organic units linked by covalent bonds or formed through reactions with metal clusters (such as Zeolites, COFs, organic Cages, MOFs, and PIMs).

Given the application of energy storage, the unique structural characteristics of these microporous materials make them attractive as ion‐conductive membranes for the following reasons [126]:

- **Structural functionality**: The structure and properties of materials can be precisely regulated by designing organic linkers, tuning metal clusters, or specifically modifying polymer frameworks, thereby endowing them with targeted functionalities. This can be achieved by, for instance, incorporating functional groups with ion‐conducting characteristics into the architecture of microporous materials to effectively enhance their ion transport efficiency.
- **Porous and open channels**: Interconnected microporous networks endow the material with excellent electrolyte wettability and retention capacity, reducing physical leakage while ensuring sufficient ion transport interfaces. Simultaneously, the uniform and appropriately sized pores can regulate lithium‐ion transport and distribution through nanoconfinement effects, thereby effectively suppressing lithium dendrite growth and side reactions, and enhancing battery safety and cycling stability.
- **High free volume and surface area**: Benefiting from the high free volume within the material, the carrier density is increased, while long‐range migration pathways for ions are formed, thereby ensuring that the membrane possesses sufficient ionic conductivity.
- **Tunable pore size**: Controllable pore size design allows the membrane to be specifically tailored according to requirements. To address the dissolution and loss of redox‐active species in battery systems such as Li─S and ORFBs, the pore size and pore structure of the membrane can be precisely regulated. This enables selective blockage of larger active material molecules or ions while ensuring ionic conductivity, thereby fundamentally mitigating capacity fade caused by the “shuttle effect”.
- **Insulating property and good stability**: The electrolyte membrane should serve a physical separation function for the electrodes to prevent battery short‐circuit issues, while it does not participate in the redox reaction process itself. In addition, the membrane should possess multiple basic stability properties, specifically including thermal stability, electrochemical stability, and mechanical stability. Different types of microporous materials provide the possibility for the design of such custom‐tailored insulating and stable membranes.

Currently, the design of ion‐conductive membranes based on emerging microporous materials is gradually penetrating the fields of energy storage and conversion. Microporous materials include Cages, COFs, MOFs, PIMs, and metal–organic polyhedra (MOP) [127]. Here, we will mainly introduce the applications of COFs, MOFs, and PIMs as battery membranes.

### 3.1 Covalent Organic Frameworks

COFs are a class of crystalline porous materials constructed from organic building units linked by strong covalent bonds. They exhibit high specific surface area, tunable pore size, and tailorable surface chemistry, demonstrating significant potential for application in battery membranes. The core of COFs preparation lies in precisely linking organic building units via covalent bonds to construct two‐dimensional or three‐dimensional crystalline networks with periodically ordered pore channels. Primary methods include solvothermal synthesis, mechanochemical synthesis, interfacial polymerization, and in situ growth. Solvothermal synthesis is a conventional approach capable of producing COF powders with high crystallinity and ordered pore structures under high temperature and pressure. However, its scalability is limited by issues such as high energy consumption, lengthy reaction times, and frequent use of toxic solvents. As a greener alternative, mechanochemical synthesis employs mechanical force to drive reactions rapidly under ambient conditions, eliminating the need for toxic solvents, though the resulting materials generally exhibit lower crystallinity. Interfacial polymerization involves conducting the polymerization reaction at the interface between two immiscible phases to form ultrathin COF membranes. This method offers advantages such as uniform film formation and controllable thickness, yet it requires stringent preparation conditions and often yields membranes with limited mechanical strength. In situ growth refers to the direct deposition of COF layers on existing substrates; however, achieving precise control over film thickness and uniformity remains a challenge.

#### 3.1.1 Ion‐Transport Mechanism

The efficient ion transport mechanisms in COF membranes primarily rely on size sieving, functionalization, and hydrogen‐bonding network conduction. The ordered pore structure and tunable topological design of COFs form continuous ion channels, which selectively sieve ions based on size differences, effectively blocking larger ions or molecules. Functionalization with charged groups introduced into the framework enhances the membrane's surface charge density, enabling selective transport of target ions through electrostatic attraction or repulsion. Additionally, functional groups within the framework form stable hydrogen‐bonding networks with water molecules, establishing efficient proton transport pathways. These mechanisms work synergistically, endowing COF membranes with both excellent ion selectivity and high conduction efficiency.

#### 3.1.2 Functional Membranes

**FIGURE 9:**
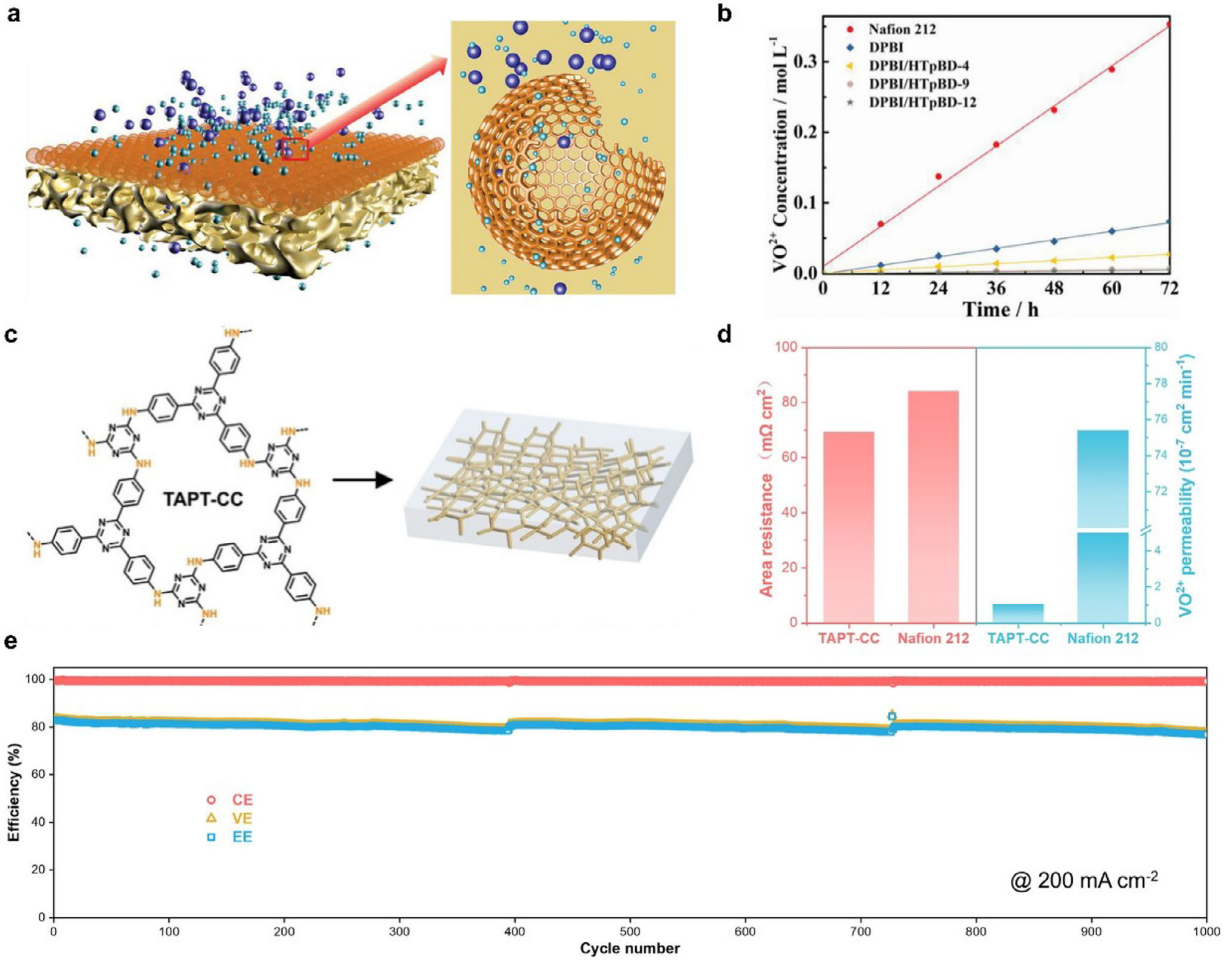
(a) Schematic diagram of the ion transport process of DPBI/HTPBD composite membrane. (b) The VO^2+^ permeability of DPBI/HTPBD composite membrane [128]. (c) Self‐standing TAPT‐CC membrane and chemical structure. (d) Comparison of VO^2+^ permeability and area resistance of the self‐standing TAPT‐CC and Nafion 212 membranes. (e) Long cycling performance of VFB equipped with self‐standing TAPT‐CC membrane at a current density of 200 mA cm^−2^ [129]. (a,b) Reproduced with permission [128]. Copyright 2022, Wiley‐VCH GmbH. (c–e) Reproduced with permission [129]. Copyright 2024, Wiley‐VCH GmbH.

Owing to their stable covalent frameworks, COFs typically exhibit excellent chemical and thermal stability, along with good compatibility with polymer matrices. Consequently, they are often employed as functional reinforcing components in composite membranes to enhance V^+^/H^+^ selectivity in VRFBs. For instance, utilizing hollow spherical COF structures as selective layers leverages their high porosity and well‐defined nanochannels to achieve efficient and selective proton transport while effectively blocking vanadium ion permeation (Figure 9a,b). This approach significantly increases the energy efficiency of the battery from 67.2% to 89.5%. Beyond morphological control, the design of pore size and functional groups in COFs is also crucial. Through in situ polymerization via nucleophilic substitution, self‐standing TAPT‐CC membranes (Figure 9c) with sub‐nanometer pore channels (4.5–6.4 Å) can be prepared. Simultaneously, the secondary amine groups within the membrane form continuous hydrogen bond networks with water molecules, collectively constructing efficient proton conduction pathways. By combining size sieving and charge repulsion mechanisms, this membrane effectively blocks vanadium ion permeation and demonstrates exceptional ion selectivity (Figure 9d). Batteries equipped with this membrane maintain stable operation for over 1000 cycles at a current density of 200 mA cm^−2^ (Figure 9e), indicating excellent long‐term cycling stability. Despite the prominent structural and functional advantages of COFs, their large‐scale fabrication remains constrained by multiple challenges, including precise control over crystallinity and pore architecture, as well as process complexity and cost. Future research should focus on the development of milder and greener synthetic strategies, coupled with rational framework design and surface functionalization, to further enhance material performance.

### 3.2 Metal–Organic Frameworks

**FIGURE 10:**
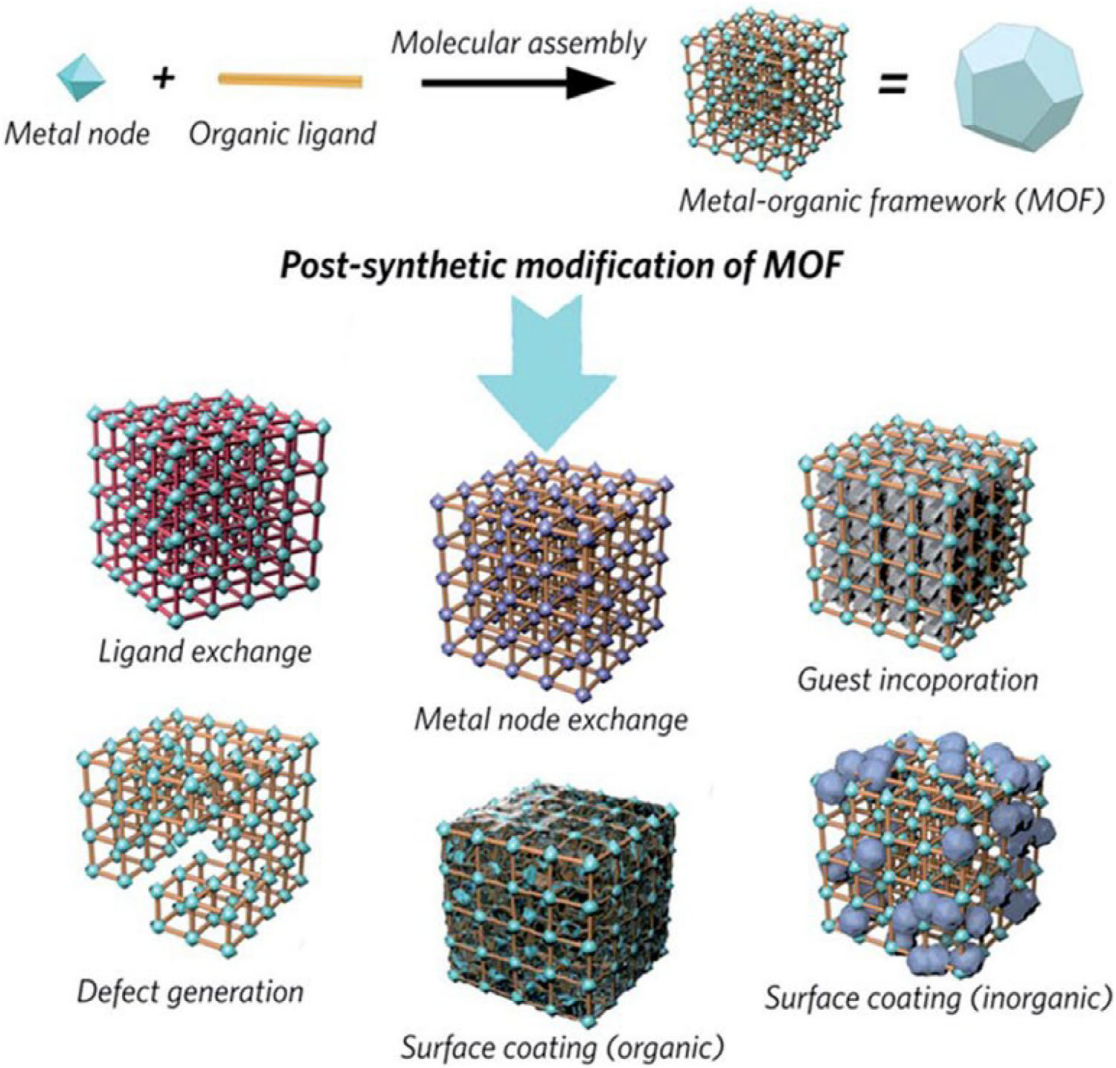
Scheme showing the typical synthesis of MOFs and relevant post‐synthetic modifications [135]. Reproduced with permission [135]. Copyright 2020, Royal Society of Chemistry.

MOFs are a class of microporous crystalline materials assembled from metal centers and organic linkers, exhibiting rich chemical diversity. Their structure demonstrates a modular nature at the molecular level, which confers a range of distinctive properties, such as ease of functionalization, tunable pore size, high free volume, customizable surface chemistry, and good physical stability [130]. Owing to the high tunability in both structure and function, MOF materials can be modified and engineered through various post‐synthetic methods, including unit exchange, guest molecule encapsulation, defect engineering, and surface coating (Figure 10). Taking defect engineering as an example, this strategy can effectively create open metal sites, functionalize pore surfaces, and enhance local porosity, which holds significant importance for separation, healthcare, catalysis, and energy applications. In the field of energy storage, membranes derived from MOFs (such as UiO‐66 [131], MIL‐101 [132], ZIF‐8 [133], and MOF‐801/MOF‐808 [134]) have been successfully applied to RFB systems. These MOF‐modified membranes primarily rely on pore‐size sieving and surface functionalization mechanisms to effectively block active species while facilitating rapid transport of charge carriers, thereby enhancing the coulombic efficiency, energy efficiency, and stability of RFBs.

Although MOFs possess unique performance advantages, their poor processability remains a bottleneck for practical applications. Currently, conventional methods for preparing MOF‐based structural materials include the use of polymer binders, blending or in situ growth of MOFs on substrates, and mechanical compression. Taking MOF/polymer composite membranes as an example, while they can enhance material flexibility and mechanical strength, issues such as limited effective loading capacity and pore blockage by the polymer binder persist [136]. Although introducing a second component can improve certain properties, it may come at the expense of gas adsorption capacity, catalytic efficiency, and uniformity of ion transport. Therefore, developing self‐supported shaping technologies for MOFs that require no additional components is crucial for overcoming application bottlenecks and unleashing their intrinsic performance potential.

#### 3.2.1 Ion‐Transport Mechanism

**FIGURE 11:**
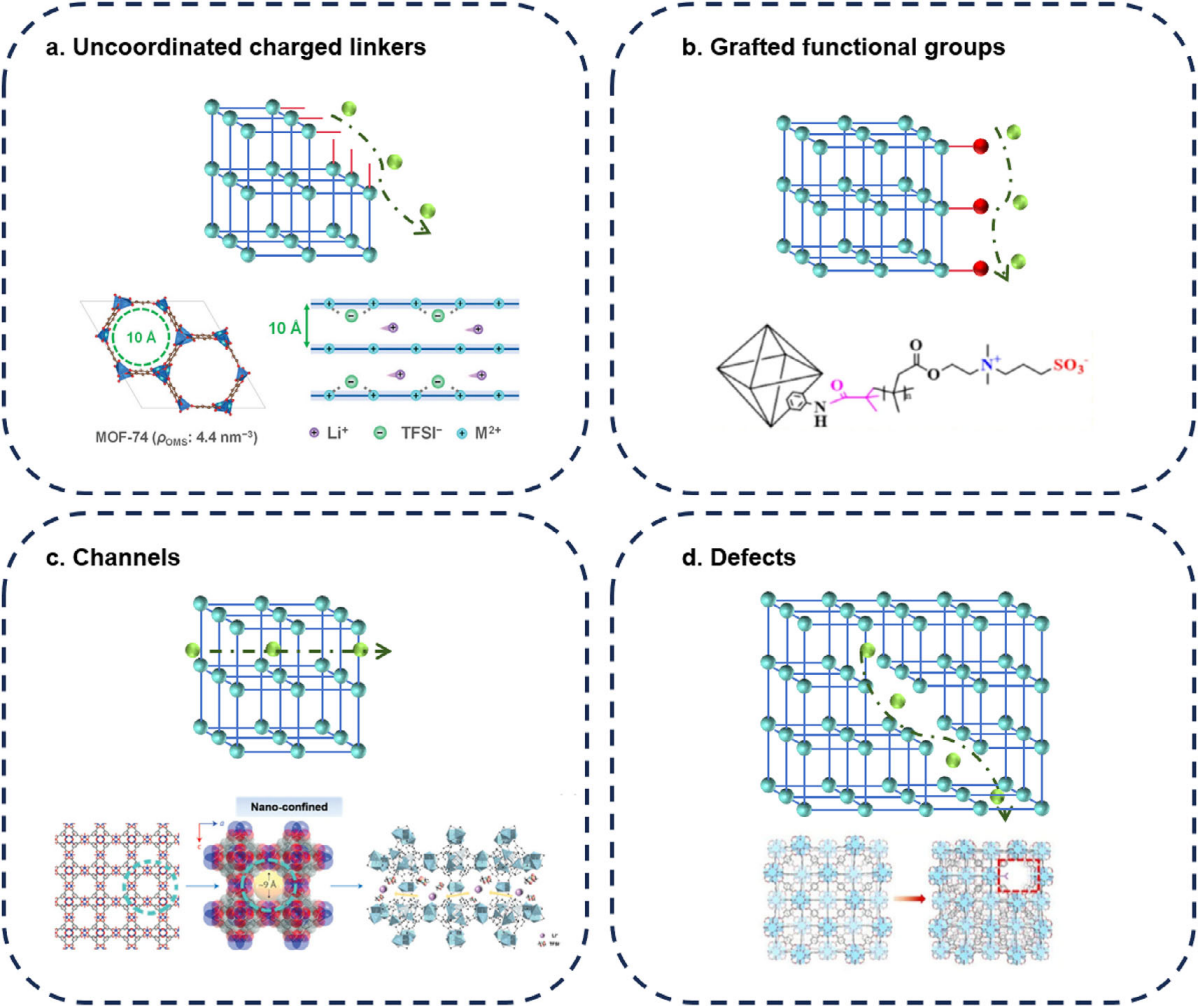
Working mechanism of ion‐transport in MOFs [137, 138, 139, 140]. Reproduced with permission [137]. Copyright 2023, Wiley‐VCH GmbH. Reproduced with the permission [138]. Copyright 2025, Elsevier B.V. Reproduced with the permission [139]. Copyright 2024, Wiley‐VCH GmbH. Reproduced with permission [140]. Copyright 2024, American Chemical Society.

A thorough understanding of its working principle (especially the ion transport mechanism) is crucial for comprehending the electrochemical properties of MOF‐based materials, and it can also provide guidance for the future development of practical materials. Based on representative findings (Figure 11), several plausible ion transport mechanisms are summarized below.

- **Uncoordinated charged linkers**: Taking typical one‐dimensional channel‐type MOFs (such as MOF‐74 [137] and Mg_2_(dobdc) [141]) as examples, these materials contain unsaturated metal sites and non‐coordinating charged groups on the framework. On the one hand, the unsaturated metal ions in the channels coordinate with anions from the electrolyte, promoting the dissociation of lithium salts, increasing the concentration of free Li^+^, and thereby facilitating lithium‐ion transport. On the other hand, the non‐coordinating charged groups can provide continuous hopping sites for Li^+^, further enhancing lithium‐ion conduction.
- **Grafted functional groups** [142, 143]: Based on the structural tailorability of MOFs, specific functional groups can be grafted onto their surfaces to construct functionalized ion transport channels. Taking UIO‐66‐PS [138] as an example, by grafting poly(sulfobetaine methacrylate) (PSBMA) onto the surface of UIO‐66‐NH_2_, both sulfonate groups and quaternary ammonium groups are simultaneously introduced, thereby constructing highly efficient ion transport channels.
- **Channels**: MOFs provide continuous and tunable pathways for ion transport due to their highly ordered sub‐nanometer channels. Taking HKUST‐1 [139] as an example, it possesses a three‐dimensionally interconnected pore network with an aperture of about 14 Å and a window size of approximately 9 Å. These sub‐nanometer channels can physically screen solvated ions, allowing only size‐matched ion–solvent complexes to enter the interior pores. Meanwhile, the geometric confinement of the channels alters the coordination environment of Li^+^, weakening its interaction with anions and thereby promoting lithium‐salt dissociation.
- **Defects**: Utilizing the internal defects of MOFs holds potential value for developing novel ion conductors. Expansion of conductive space via defect networks: Interconnected point defects form an extended defect network, enlarging the pore size (e.g., the UiO‐66‐Dx series materials increased from 11 to 18.2 Å) [140], providing more accommodation space and continuous migration channels for charge carriers. Electrostatic correlation between defects and ionic conductivity: The introduction of defects is often accompanied by unsaturated metal sites and uncoordinated ligands (such as dangling ─COO^−^ groups). These sites efficiently adsorb and stabilize Li^+^ through electrostatic interactions, forming low‐energy‐barrier hopping sites.

#### 3.2.2 Functional Membranes

**FIGURE 12:**
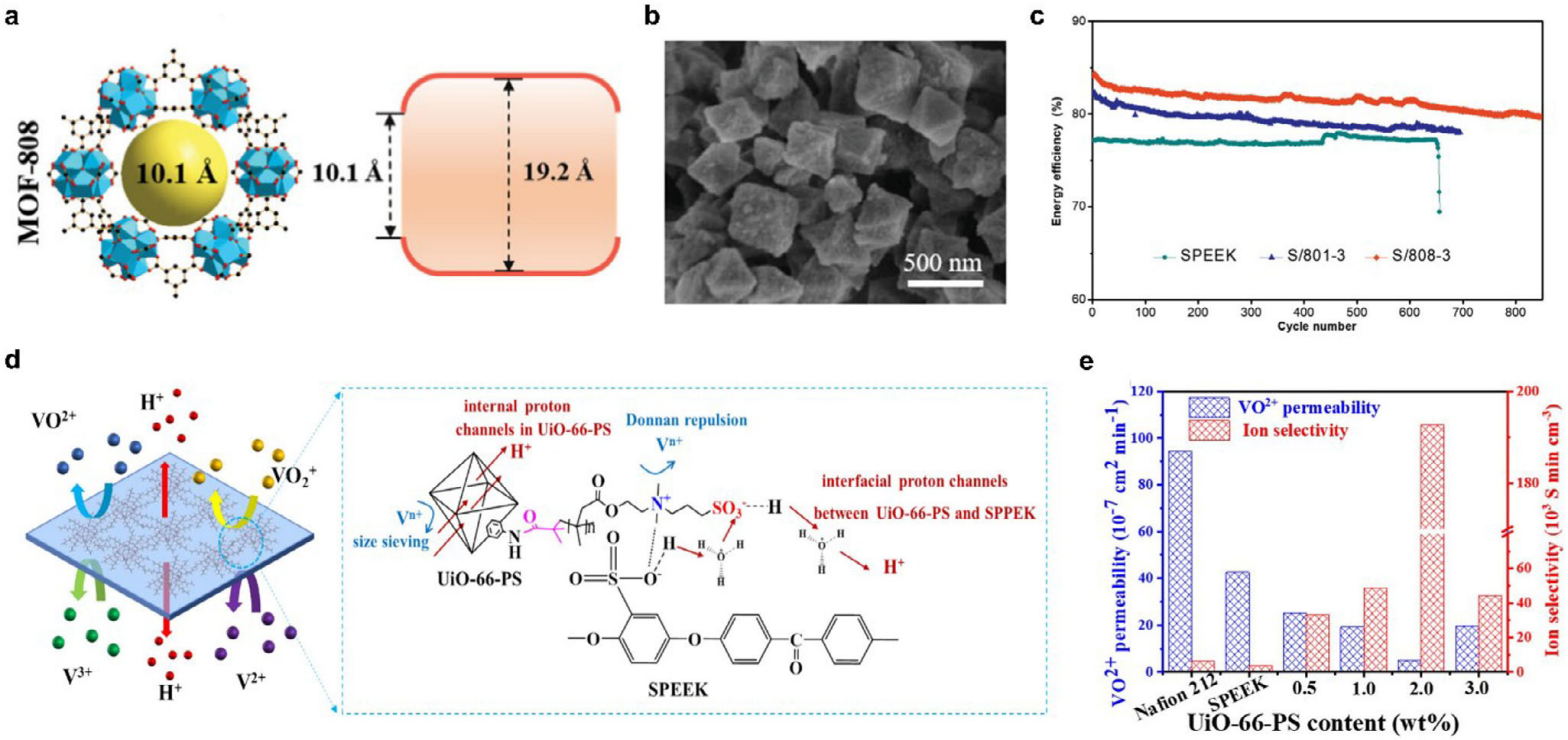
(a) Window and cavity size of MOF‐808. (b) SEM images of MOF‐808 powders. (c) Cycle performance of VRFBs equipped with SPEEK, S/801‐3, and S/808‐3 at 120 mA cm^−2^ [144]. (d) Schematic illustration of the synergistic mechanism for ion‐selective transport in S/UiO‐66‐PS membranes. (e) VO^2+^ permeability and ion selectivity [84]. (a–c) Reproduced with permission [144]. Copyright 2021, Wiley‐VCH GmbH. (d,e) Reproduced with permission [84]. Copyright 2025, Elsevier B.V.

In VRFBs, MOFs can construct proton‐selective transport channels through their tunable pore structures and surface chemical properties via a molecular sieving effect, thereby significantly enhancing the ion selectivity and conductivity of the membrane. For instance, incorporating MOF‐808 (Figure 12a) as a functional filler into a sulfonated SPEEK matrix effectively suppresses vanadium ion permeation while improving proton conductivity [144]. This leads to a notable increase in both the coulombic efficiency and energy efficiency of the battery (Figure 12c). Similarly, defect‐free UiO‐66/UiO‐67 composite membranes have been fabricated on polymer substrates via an in situ binder‐controlled restrained second‐growth method. Their ordered pore structures effectively regulate ion transport selectivity, and when applied in a zinc‐iodine flow battery, the coulombic efficiency was increased from 88.4% to 94.5%. This further demonstrates the potential of MOF materials in enhancing ion selectivity and battery performance [131]. Furthermore, surface functionalization of MOF materials can optimize their dispersibility within the polymer matrix and interfacial transport behavior. For example, UiO‐66‐PS nanomaterials modified with zwitterionic polymers form continuous and efficient proton transport channels within the SPEEK membrane, increasing the ion selectivity of the composite membrane to 49 times that of the pure SPEEK membrane (Figure 12d,e). The VRFB equipped with this membrane achieves a high energy efficiency of 87.1% at a current density of 100 mA cm^−2^, demonstrating the significant advantages of functionalized MOFs in improving the overall battery performance [84]. Although MOFs show significant potential in the field of energy storage, their large‐scale application still faces challenges such as insufficient chemical stability, a tendency to agglomerate, low mechanical strength, and poor interfacial compatibility with polymers. Future research should focus on selecting metal nodes with high stability, enhancing stability through surface functionalization, and adopting in situ growth techniques to improve interfacial adhesion and enhance the overall performance of membrane materials, thereby accelerating their practical application in advanced battery systems.

### 3.3 Polymers of Intrinsic Microporosity

PIMs are a class of amorphous polymers characterized by rigid and contorted backbone structures, first proposed by N.B. McKeown and P.M. Budd in 2003 [145]. Their polymer chains exhibit restricted conformational flexibility and cannot pack efficiently due to their highly contorted skeletons, resulting in low rotational freedom along the main chain. PIMs possess excellent organic solvent solubility, high microporosity and specific surface area, narrow pore size distribution, and good thermal stability. These properties make them widely applicable in fields such as gas separation [146, 147], liquid separation [148], hydrogen storage [149], and energy storage technologies [150].

The characteristics of PIMs are as follows:

- **Solution‐processability**: Unlike traditional “insoluble” porous materials such as activated carbon or zeolites, PIMs are soluble in organic solvents and can be readily processed into films [146, 147], fibers [151], or porous particles through solution‐processable techniques such as casting and spin‐coating, thereby enabling their broad application in separation, storage, and energy‐related fields.
- **High microporosity and free volume**: PIMs possess rigid and contorted polymer chain conformations that prevent close packing, thereby creating a large number of continuous, inherent micropores within the material. This characteristic endows PIMs with a high specific surface area and free volume. Their abundant microporous channels and cavities enable outstanding performance potential in applications such as membrane‐based gas separation and adsorption.
- **Narrow Pore Size Distribution**: Benefiting from their rigid backbone and specific molecular configurations, PIMs can form inherent cavities of uniform size, leading to a concentrated sub‐nanometer pore size distribution. This enables them to selectively transport small molecules while effectively blocking larger ones.
- **Tunable Functionality**: The diverse chemical compositions of PIMs often contain reactive groups such as cyano groups, which provide convenience for subsequent chemical modifications. Through modification methods such as amidation, cross‐linking, carboxylation, and amination, the pore size distribution, surface properties, hydrophilicity/hydrophobicity, and other specific functionalities of the material can be further regulated.

#### 3.3.1 Ion‐Transport Mechanism

**FIGURE 13:**
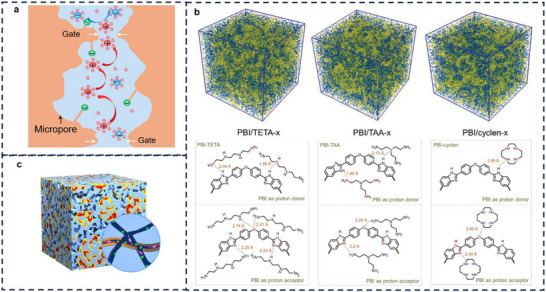
(a) Scheme of the ion transport within functional groups grafted micropores. (b) Supramolecular interactions between PBI and amines with different topologies [71]. (c) confined microphase separation with intrinsic ultra‐micropore [152]. (b) Reproduced with permission [71]. Copyright 2023, Wiley‐VCH GmbH. (c) Reproduced with permission [152]. Copyright 2025, Elsevier Inc.

The application of PIMs‐based membranes in energy storage systems has become a research hotspot. Understanding their working mechanisms, particularly the ion transport mechanism, is crucial as it can provide guidance for developing novel ion‐conductive materials. In general, the incorporation of hydrophilic ionic groups into membrane materials markedly improves ion transport [153]. Representative examples include anionic moieties (─SO_3_^−^, ─PO_3_^2−^, ─PO_3_H^−^, ─COO^−^) and cationic groups (─NH_3_^+^, ─NR_2_H^+^, ─NR_3_^+^). These functional groups serve as active sites that facilitate charge carrier conduction(Figure 13a). However, functionalized membranes without microstructural regulation often suffer from severe swelling. The expanded ion transport channels resulting from swelling can lead to a loss of selectivity toward large‐sized active species [63]. To address this issue, researchers have introduced phase‐separated microstructures into polymer frameworks to suppress membrane swelling and construct narrow‐channel ion transport pathways. Through molecular design, more precise topological control of the phase‐separation structure can be achieved, thereby optimizing channel size and connectivity. For instance, by non‑covalently grafting amine side chains with different topological structures (linear, branched, cyclic) onto the PBI backbone, a “pseudo‑nanophase‑separation” structure has been successfully constructed (Figure 13b) [71]. This approach enables accurate regulation of the size and connectivity of hydrophilic channels. The resulting structure not only effectively suppresses excessive swelling of the membrane under hydrated conditions but also forms continuous sub‑nanometer hydrophilic channels. Consequently, high proton conductivity is achieved while vanadium‑ion permeability is significantly reduced, demonstrating excellent selectivity.

In addition, hydrophilic PIMs may themselves possess an interconnected microporous structure, which can work in conjunction with ion‐conducting groups to facilitate carrier transport. Since their inherent micropores can effectively suppress the transmembrane permeation of redox‐active species, the development of PIMs with both ionic conductivity and selectivity may not require sophisticated design of hydrophilic domains and narrow ionic channels. Similarly, hydrophilic PIMs incorporating Tröger’ s base (TB) functional structures can accommodate charge carriers through their interconnected micropores. For instance, by integrating a semi‐rigid Tröger’ s base (TB) backbone with flexible branched architectures, ultramicropores and microphase‐separated ion channels can be combined within the membrane (Figure 13c) [152]. This unique dual‐ion‐channel system features size‐restricted transport pathways, enabling smooth and highly selective ion transport.

#### 3.3.2 Ion‐Selective Membranes for RFBs

**FIGURE 14:**
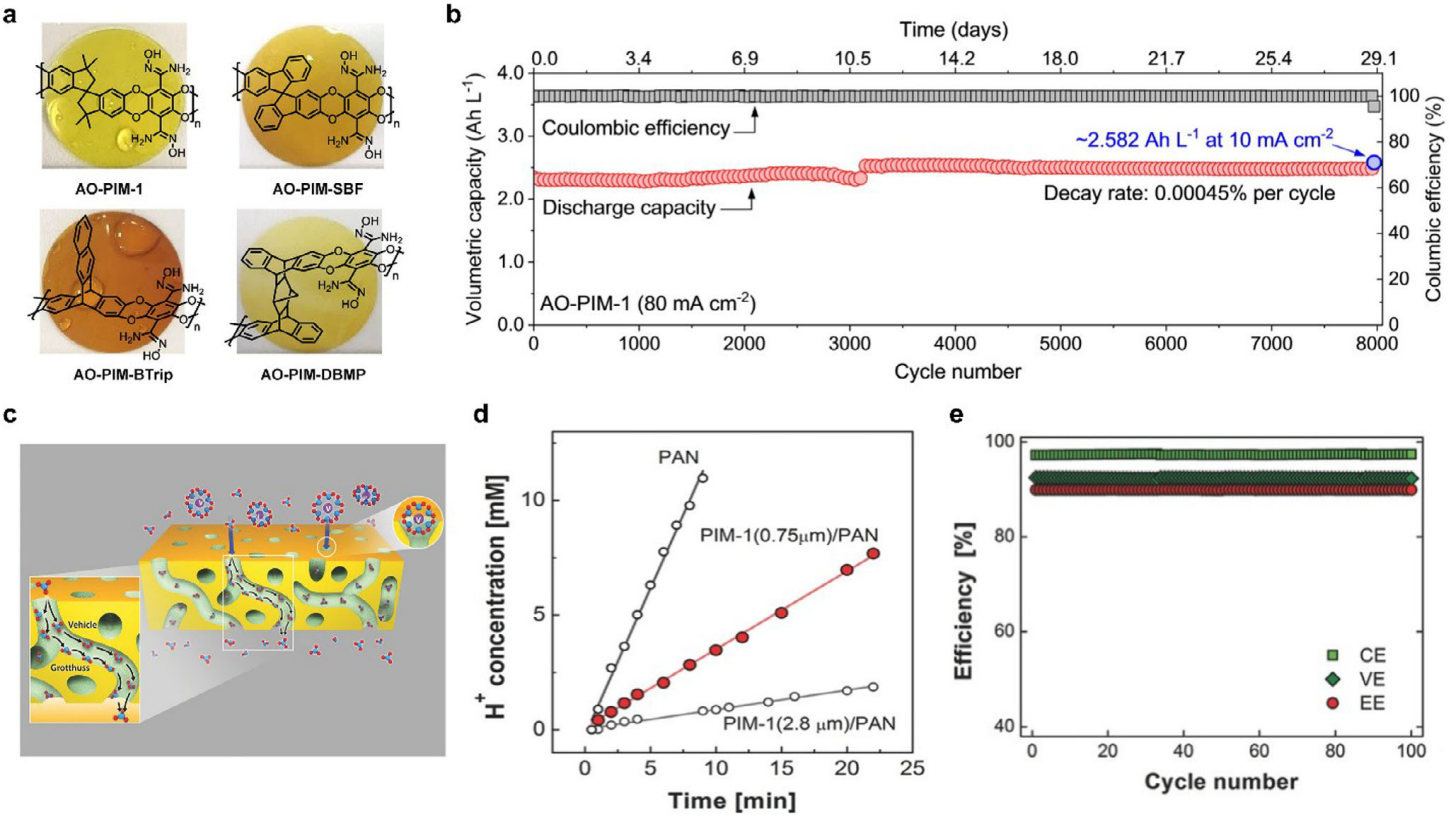
(a) Chemical structures and photographs of corresponding 50‐µm‐thick self‐standing membranes of AO‐PIMs including AO‐PIM‐1, AO‐PIM‐SBF, AO‐PIM‐BTrip, and AO‐PIM‐DBMP, respectively. (b) AO‐PIM‐1 membranes in 0.1 m 2,6‐DPPAQ|K4Fe(CN)6 RFB cells at pH=9 and a current density of 80 mA cm^−2^ [154]. (c) Molecular structure of PIM‐1 and its working principle for the proton conduction. (d) the H+ permeation rates when using PAN without PIM‐1, 0.75, and 2.8 µm thickness PIM‐1/PAN membranes, respectively. (e) Cycling performance of VRFBs using PIM‐1 membranes [155]. (a,b) Reproduced with permission [154]. Copyright 2022, Wiley‐VCH GmbH. (c–e) Reproduced with permission [155]. Copyright 2016, Wiley‐VCH GmbH.

PIMs demonstrate broad application prospects in IEMs and multiphase interface design for RFBs due to their high specific surface area, tunable microporous structure, and excellent solution processability. The microporous channels of PIMs can be precisely regulated through molecular design strategies, such as introducing specific functional groups onto the polymer backbone and optimizing their topological arrangement (Figure 14a), which enables the formation of non‐close‐packed structures in the solid state. This allows fine control over microporous geometry, size distribution, and mass transport pathways, thereby further enhancing ion selectivity. Membranes fabricated from amidoxime‐functionalized PIMs (AO‐PIMs) [154] based on this strategy exhibit outstanding long‐term cycling performance in aqueous organic flow batteries (Figure 14b). On the other hand, hydrophobic PIMs (e.g., PIM‐1) effectively restrict excessive swelling in acidic electrolytes (Figure 14c), where their narrow pore channels mitigate vanadium ion (e.g., VO^2+^) permeation while facilitating efficient proton transport via the Grotthuss mechanism, significantly improving H^+^/V^+^ selectivity and enhancing battery operational stability (Figure 14d,e) [155]. Additionally, incorporating rigid hydrophobic pendant groups into amidoxime‐functionalized PIM enables precise control of hydrated pore gates at the sub‑nanometer scale (5 Å), which simultaneously achieves high ionic conductivity and extremely low permeability of redox‑active species. Flow batteries employing this membrane exhibit a capacity decay rate as low as 0.014% per day [97]. To further improve the overall performance of PIM membranes, researchers have incorporated functional groups such as sulfonic acid and amidoxime into the PIM backbone, developing membranes like SPX‐BP and AquaPIM suitable for alkaline systems [125, 156, 157]. These functionalized PIM membranes maintain high ion selectivity while demonstrating enhanced chemical stability and the ability to discriminate between different active species. In summary, PIMs demonstrate significant advantages in the field of flow battery membranes through functionalization strategies. However, their industrialization is hindered by performance decay due to physical aging, the inherent trade‐off between permeability and selectivity, and the inadequate processability and long‐term stability of certain materials. Future efforts will focus on research in molecular design, structural hybridization, and process innovation—such as precisely tuning the microporous structure through the combination of functional groups and mild cross‐linking—to balance ion selectivity and conductivity.

## 4 Challenges and Prospects

This review systematically summarizes recent advances in membranes for RFBs, with a focus on the structural characteristics, performance requirements, and underlying mechanisms governing their influence on overall battery performance. The study highlights that membranes, as core components of RFBs, play an indispensable role in regulating ion‐selective transport, maintaining electrode interface stability, and ensuring long‐term cycling durability. However, the development of high‐performance battery membranes still faces multi‐faceted challenges in balancing selectivity, conductivity, cost, and stability, calling for breakthroughs in material innovation and structural design.

Although PFSA membranes (e.g., Nafion) remain the best‐performing membrane materials in current RFB systems, their limitations—such as low selectivity toward redox‐active species, high cost, and dependence on limited resources—hinder large‐scale application. Non‐ionic exchange membranes and porous membranes (e.g., PBI) offer improved selectivity but often at the expense of ionic conductivity. Emerging microporous materials, including COFs, PIMs, and MOFs, provide new opportunities for designing highly efficient and selective membranes due to their high specific surface area, tunable pore structures, and tailorable surface chemistry. Nevertheless, these materials still face multiple challenges in practical battery environments.

To achieve comprehensive performance improvements in membrane materials, future research should focus on the following directions:

1. Developing separation strategies based on multi‐mechanistic synergy, such as incorporating charged functional groups into microporous structures to combine size exclusion and Donnan exclusion effects for enhanced ion selectivity.
2. Constructing interconnected sub‐nanometer pore channels to shorten ion transport pathways without compromising selectivity, thereby improving conductivity.
3. Enhancing mechanical and chemical stability through defect engineering, composite structure design, and novel ligand incorporation to extend membrane service life under harsh electrochemical conditions.
4. Promoting systematic matching studies between membrane materials and redox couples to establish structure–property–performance relationships under realistic operating conditions, enabling the rational design of next‐generation membranes.

## Conflicts of Interest

The authors declare no conflict of interest.

## Data Availability

The authors have nothing to report.
